# Development of a vibration-damping, sound-insulating, and heat-insulating porous sphere foam system and its application in green buildings

**DOI:** 10.1038/s41598-024-65025-0

**Published:** 2024-06-20

**Authors:** Shi Hua, Moses Oyaro Okello, Jian Zhang

**Affiliations:** School of Ship and Ocean Engineering, Jiangsu University of Science and Technology, Zhenjiang, 212003 Jiangsu China

**Keywords:** Vibration damping and acoustic insulation porous ball foam system, Airborne sound insulation performance, Impact sound insulation performance, Thermal resistance, Engineering, Civil engineering, Materials science, Structural materials, Techniques and instrumentation

## Abstract

With the development of green buildings, people pay more attention to the quality of the indoor sound environment. The air sound insulation performance of floors and exterior walls plays a key role in today's green buildings. The thermal performance of the enclosure structure's floor and exterior wall heat transfer resistance is an important factor in reducing building carbon emissions in green buildings. The aim of this paper is to study the efficiency of the acoustic and thermal insulation of a foaming system with porous carbon balls and the combination of different structural ways of construction boards and external walls. The acoustic and thermal parameters of different sound insulation and thermal insulation systems designed with porous carbon sphere foam and inserted into the floors and exterior walls are compared to highlight the optimal structure. The theoretical and experimental tests showed that to improve the sound insulation performance of the floor, a sound insulation system needs to be placed on the surface of the floor in contact with the impact object and inlaid in the vertical gap in contact with the floor and the wall. Furthermore, it has been determined that the surface of the foam particle acoustic ball with micropores has good sound absorption performance. Finally, the high-quality building thermal insulation material with low thermal conductivity in any combination with the floor slabs and the external wall structure improves the thermal insulation performance.

## Introduction

With the massive development of green buildings around the world, the building acoustic environment has become more and more highly valued and is also used as an important assessment factor for evaluating the grade of green buildings. In order to bring a quiet and comfortable living environment to all new green buildings, all new buildings have a special chapter on green building design, which has high requirements for the acoustic performance of external walls and floor slabs and heat transfer resistance. Professor Kang Jian of the UK has done a lot of research in the field of reducing urban acoustic environment noise, conducted a comprehensive study of noise reduction from the perspective of the principle of noise reduction, used the block acoustic model to predict the noise situation, verified through experiments, and put forward optimisation proposals for noise reduction in the built environment^1^. Liu Xiaotu of Southeast University researched and proposed residential sound environment indicators and put forward new research theories for improving the residential sound environment^2^. In terms of energy savings in the built environment, China has absorbed advanced energy-saving building development experience and energy-saving technology from developed countries. By 2022, the energy-saving effect of new green buildings had been improved by 30% compared with 2015, and the energy-saving standards in some developed areas were close to the energy-saving standards of developed countries in Europe and the United States^3–5^ (Research on energy-saving design of ultra-low-energy residential buildings in cold regions). At present, most domestic and foreign researchers use the "floating floor sound insulation method", that is, using flexible materials laid on the upper part of the floor to improve the sound insulation performance of the floor. But after the experiments of researchers such as Shi Hua^6^, it was proved that the "floating floor sound insulation method" can only improve the impact sound insulation performance of the floor and not significantly improve the airborne sound insulation performance of the floor. However, it has been proven that the "floating floor insulation method" can only improve the sound insulation performance of the floor slab for impact sound, and the improvement of the sound insulation performance of the floor slab for airborne sound is not obvious, which is due to the lack of sound-absorbing materials in the flexible materials. After market research and study (1) Certain insulation materials such as foamed silica gel panels, although they have the advantages of being soft, non-flammable, and moisture-proof, their sound insulation performance is general, and they may not be able to effectively block the propagation of sound; (2) Certain insulation materials such as aluminium foil composite insulation materials, the composite layer of which may be hygroscopic, and this will affect the material's sound insulation performance; (3) The aluminium foil composite insulation materials may reflect a large amount of energy back to the Diesel engine and adjacent lines, which is not only detrimental to heat dissipation, but also may accelerate the embrittlement of the line, thus affecting the sound insulation effect; (4) The compressive strength of the sound insulation material may affect its thermal insulation performance. For example, the different elastic compression modulus of nano rubber-plastic temperature sound insulation boards and inorganic thermal insulation sound insulation mortar will affect their thermal insulation effect. The vibration damping, sound insulation and thermal insulation porous ball foam system designed in this paper can have a double usage for both sound insulation and thermal insulation under scientific design and construction^7–12^. In order to demonstrate the acoustic and thermal insulation properties of the system, this paper designs an acoustic insulation, thermal insulation, carbon porous ball foaming system, and the combination of various structural modes of building slabs and external walls and tests the acoustic insulation performance of the slabs and external walls, heat transfer resistance, and other changes in the law on the spot. Then compares and analyses the experimental results of the combination of the several modes to prove the feasibility of the designed acoustic insulation and thermal insulation carbon porous ball foaming system in the application of building slabs and external walls. Finally, the results are compared and analysed to prove the feasibility of the designed acoustic insulation and thermal insulation carbon porous sphere foam system in the application of building floors and external walls, and the results have certain reference values for the improvement of the acoustic and thermal performance of the floor and external wall in future green buildings.

## Materials and methods

### Design and preparation of a sound insulation and thermal insulation carbon porous ball foaming system

The carbon raw material of this design is activated carbon fibre, which is an ideal multifunctional material with a large specific surface area and a developed microporous structure^13^, which possesses the characteristics of heat insulation and fire prevention. At the same time, due to the three-dimensional mesh arrangement of fibres inside the activated carbon fibre mat, there are a large number of interconnecting pores between fibres, which meets the structural requirements of sound absorption of porous materials, and it is also a kind of excellent porous acoustic absorption material^14^. Therefore, experts at home and abroad have carried out extensive and in-depth research on activated carbon fibre materials, which have been widely used in the fields of environmental protection, military industry, medicine, chemical industry, food, electronics, and so on^15–20^.

The acoustic insulation and thermal insulation carbon porous ball foam system designed in this paper is divided into a combination of base acoustic insulation board and foam particle acoustic ball. The specific preparation process is as follows: (1) The base acoustic insulation board is provided with a number of uniformly arranged circular holes, circular holes nested in the foam particle acoustic ball, as shown in Fig. 2. The sound-absorbing (acoustic) insulation board is composed of the following material components: 90–105 parts of HB polymer cement-based JS-II type, 4–6 parts of carbon powder, 2–3 parts of wood chip powder, 4–6 parts of 9003–35-4 high-temperature-resistant phenol–formaldehyde resin, 6–7 parts of high-temperature-resistant glass beads, 9–11 parts of ultrafine inorganic rock wool fibres, 145–155 parts of dust-free distilled water, and 48–52 parts of AC acoustic insulation material Foaming agent. (2) The sound-absorbing sphere of foamed particles is composed of the following components: 100 parts of HB polymer cement-based JS-II, 5 parts of carbon powder, 2 parts of wood dust, 5 parts of 9003-35-4 high-temperature-resistant phenolic resin, 6/7 parts of high-temperature-resistant glass beads, 10 parts of ultrafine inorganic rock wool fibres, 150 parts of dust-free distilled water, and 50 parts of AC sound-insulating material foaming agent.

The raw materials mentioned in (1) and (2) are fed into a pulper and stirred uniformly to form a paste. The foaming agent is added to make cement foaming agent, and the cement foaming agent is poured into a model of the corresponding size and put into a curing room for hardening to form a raw solid. Among them, the foam particle acoustic ball needs to wet cut the raw solid, the cutting unit is six peeling surfaces, five peeling surfaces, or three peeling surfaces and the cut raw solid is cured and moisturised for 7–10 days until it naturally dries into a finished product. At the same time, the base acoustic insulation boards need to be rolled and pressed into thin boards of the required thickness.

(3) Carbon fibre wave mesh inlay technology is inserted to fix the "small ball": the use of direct 1–1.5 mm carbon fibre, divided into four areas of the "wave" shape diagonally fixed foam particles acoustic ball in the aluminium fibre acoustic panel cavity, and finally with carbon fibre wave mesh It is fixed as shown in Fig. 2.

**Figure 1:**
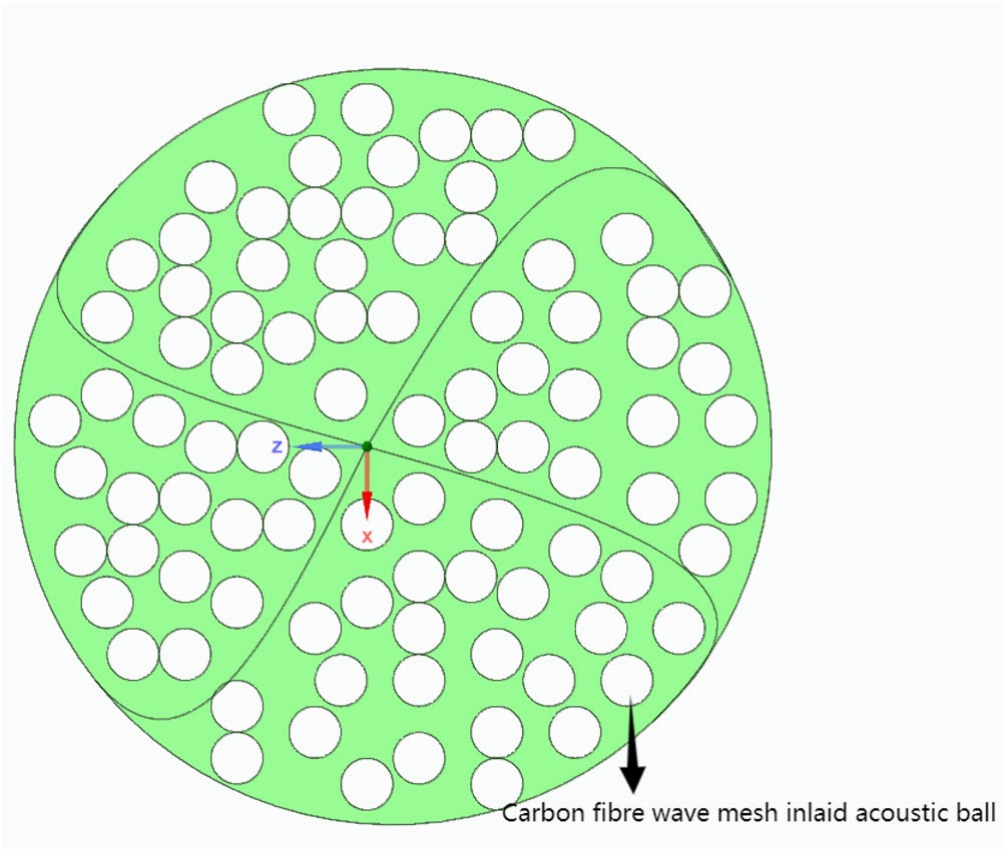
Carbon fiber wave mesh inlaid porous acoustic sphere cross-section.

**Figure 2:**
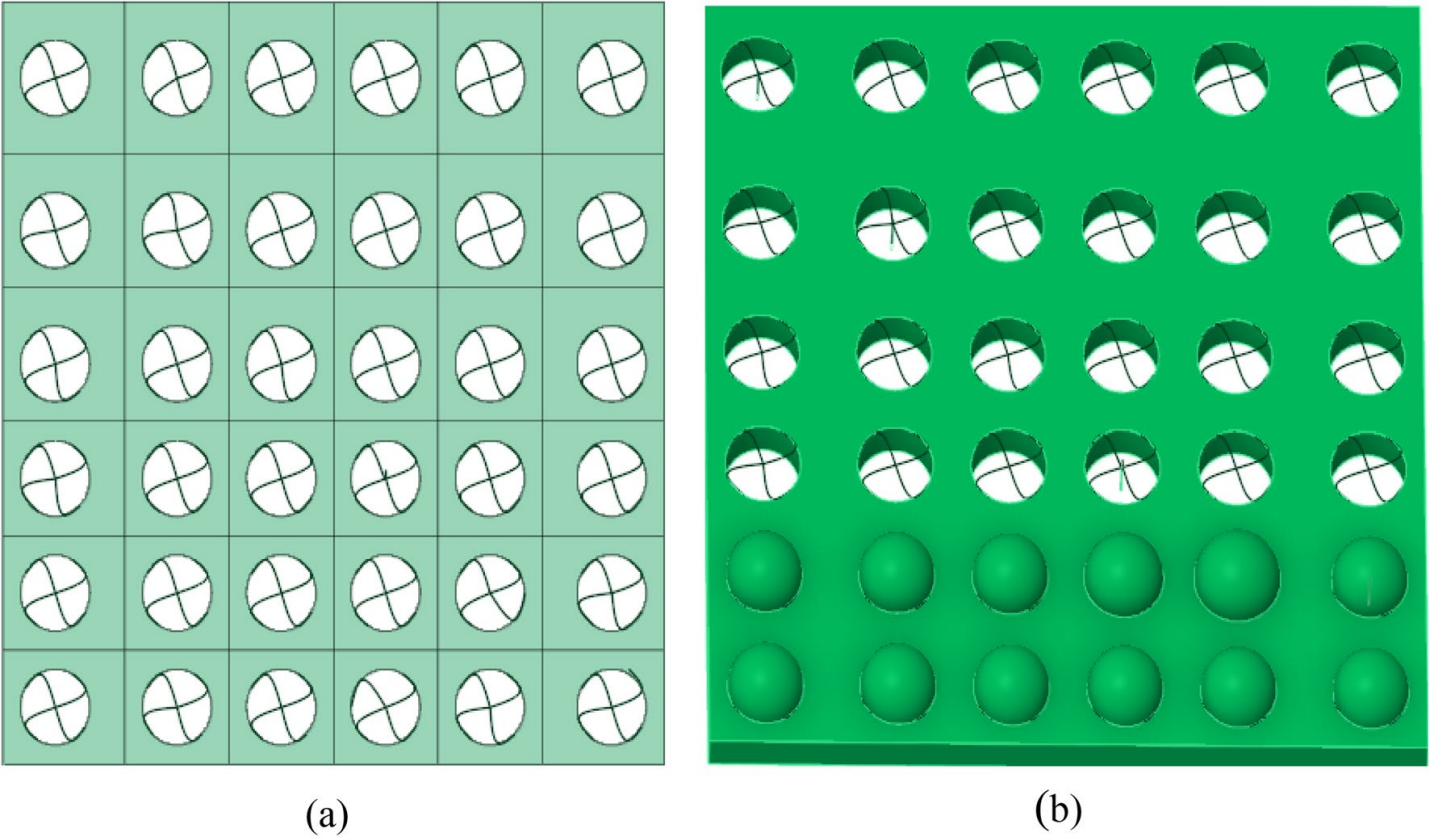
Model of the sound-insulating and thermal-insulating carbon fibre porous ball foam system: (**a**) Figure shows a plan view of the fixed distribution of the carbon fibre wave network, and (**b**) Figure shows a cross-section of the location of the sound-absorbing 'blob' inlay.

The above sound insulation, thermal insulation carbon porous ball foaming system foam particles acoustic ball consist of the following mass components: HB polymer cement-based JS-II type, activated carbon fibre, wood chip powder, high-temperature-resistant phenol–formaldehyde resin, high-temperature-resistant glass beads, ultrafine inorganic rock wool fibres, dust-free distilled water, and high foaming elastic material. The structural schematic diagram is shown in Figs. 1 and 2:

Analysis of acoustic principles: (1) The surface of the foam particle acoustic ball has microscopic holes, and these holes are the key mechanism for sound absorption. When the noise hits these tiny holes, it will produce a frequency collision and convert the acoustic energy into thermal energy. In addition, the irregular bonding of particles inside the foam particle acoustic sphere results in the presence of a number of microscopic pores around the tiny pores, which increases the viscous resistance of the air inside the pores. When sound waves enter the interior of the acoustic sphere material, they propagate along the pores on the surface of the blade, causing vibration of the air molecules in the voids. Due to the viscous resistance of the air and the friction between the air molecules and the pore walls, the acoustic energy is converted into thermal energy and lost. (2) Sound insulation, thermal insulation carbon porous ball foam system due to the addition of high foaming elastic materials, black/grey carbon fibre raw materials, composite material expansion glass beads, and lightweight particles. These materials greatly increase the value of the modulus of elasticity of the final moulding system materials (Pa). Elastic modulus refers to the elastic modulus of the system under compression. After our test, the compressive elastic modulus (Pa) of the insulation system will have the following changes due to different thickness: (1) When the material thickness of the insulation system is less than 20 mm, the compressive elastic modulus can be less than 0.5 MPa. (2)When the material thickness of the insulation system is greater than or equal to 20 mm, the compressive elastic modulus can be less than or equal to 1.2 MPa. So if it is laid in the floor on the grass-roots level and is formed with the ground floor between the elastic core, the ground floor impact vibration has a vibration damping. If it is laid on the floor slab, it will form an elastic core with the ground surface layer, which has a vibration damping effect on the impact vibration generated by the ground surface layer and reduces the vibration noise radiated from the grass-roots floor slab to the downstairs, thus achieving the purpose of improving the sound isolation performance of the floor slab.

Analysis of energy-saving principles: HB polymer cement-based JS-II type, activated carbon fibre, wood chip powder, 9003-35-4 high-temperature phenolic resin, high-temperature glass beads, ultra-fine inorganic rock wool fibre, high-foam elastic material, etc. selected by the system are all high-quality building thermal insulation materials with low thermal conductivity, which effectively enhances their thermal insulation performance.

(3) Preparation method and innovations: (a) The porous ball foaming system is composed of the following components in mass parts: 100 parts of HB polymer cement-based JS-II, 5 parts of carbon powder, 2 parts of wood chip powder, 5 parts of 9003-35-4 high-temperature-resistant phenol–formaldehyde resin, 6.7 parts of high-temperature-resistant glass beads, 10 parts of ultra-fine inorganic rock wool fibres, 150 parts of dust-free distilled water, and 50 parts of AC acoustic insulating material foaming agent. (b) The preparation process of said porous ball foaming system is as follows: sending the above formulated amount of cement, carbon powder, wood chip powder, phenolic resin, glass beads, rock wool fibre and water to a pulper and mixing them evenly until a paste is formed, adding foaming agent to make a cement foaming agent, pouring the cement foaming agent into the model and placing it into a curing chamber for hardening to form a raw embryo; cutting the raw embryo wet, with a cutting unit of four peeling surfaces, and after cutting the raw embryo Cured and moisturised for 7–10 days until naturally drying to a finished product. The cutting unit with four peeling surfaces can make the acoustic sphere multi-faceted and unconnected, preventing the generation of sphere resonance in the process of absorbing acoustic noise; it can also form more acoustic pores, absorbing more noise from the outside world. The strength of the material can be better guaranteed by wet-cutting the raw embryo and cutting it into multiple units. From a mechanical point of view, the wet-cutting process ensures that the physical properties of the material, such as the modulus of elasticity and yield strength, have better strength behaviour after drying. At the same time, cutting into multiple units also allows the material to be segmented when subjected to external forces, reducing the occurrence of physical damage to the material from external forces, such as fracture or peeling. Therefore, wet-cutting and cutting raw blanks into multiple units is a very effective measure to improve material performance and reduce damage. (c) The use of carbon fibre wave mesh covering the foam particle acoustic ball increases the roughness of the surface of the foam particle acoustic ball, improves the corrosion and fatigue resistance of the foam particle acoustic ball, and extends its service life. At the same time, the surface of the foam particle acoustic ball is divided into several areas by the carbon fibre with a diameter of 1–1.5 mm. And at the two ends of the diameter, the carbon fibre is fixed in the round holes of the aluminium fibre acoustic panel, which is from the perspective of structural vibration, making the foam particle acoustic ball not "resonate" in the outdoor wind and the effect of uncertain external forces. This is from the perspective of structural vibration. (d) In the preparation of the foamed particle acoustic spheres, the components played the following roles: HB polymer cement-based JS-II type was used as the main base material to provide the structure and sturdiness of the spheres; Carbon powder increased the acoustic performance of the acoustic spheres to reduce noise propagation by absorbing and dissipating acoustic energy; Wood chip powder provided a lightweight filler to reduce the weight of the spheres and increase their acoustic absorption effect; 9003-35-4 high-temperature resistant phenolic resin is used as an adhesive to firmly fix the various components together, which enhances the stability and durability of the sphere; High-temperature resistant glass beads have good thermal insulation properties, which can reduce the heat conduction inside the sphere and improve the heat preservation effect; Ultrafine inorganic rock wool fibres increase the acoustic performance of the acoustic sphere, which reduces ambient noise by capturing and attenuating the noise; Dust free distilled water is used as a solvent and mixing medium to adjust the viscosity and fluidity of the material, which facilitates mixing and preparation; AC acoustic insulation material foaming agent is used to increase the porosity of the acoustic sphere and generate bubble structure to improve the acoustic effect.

### Innovations in the development of porous sphere foaming systems

**Figure 3:**
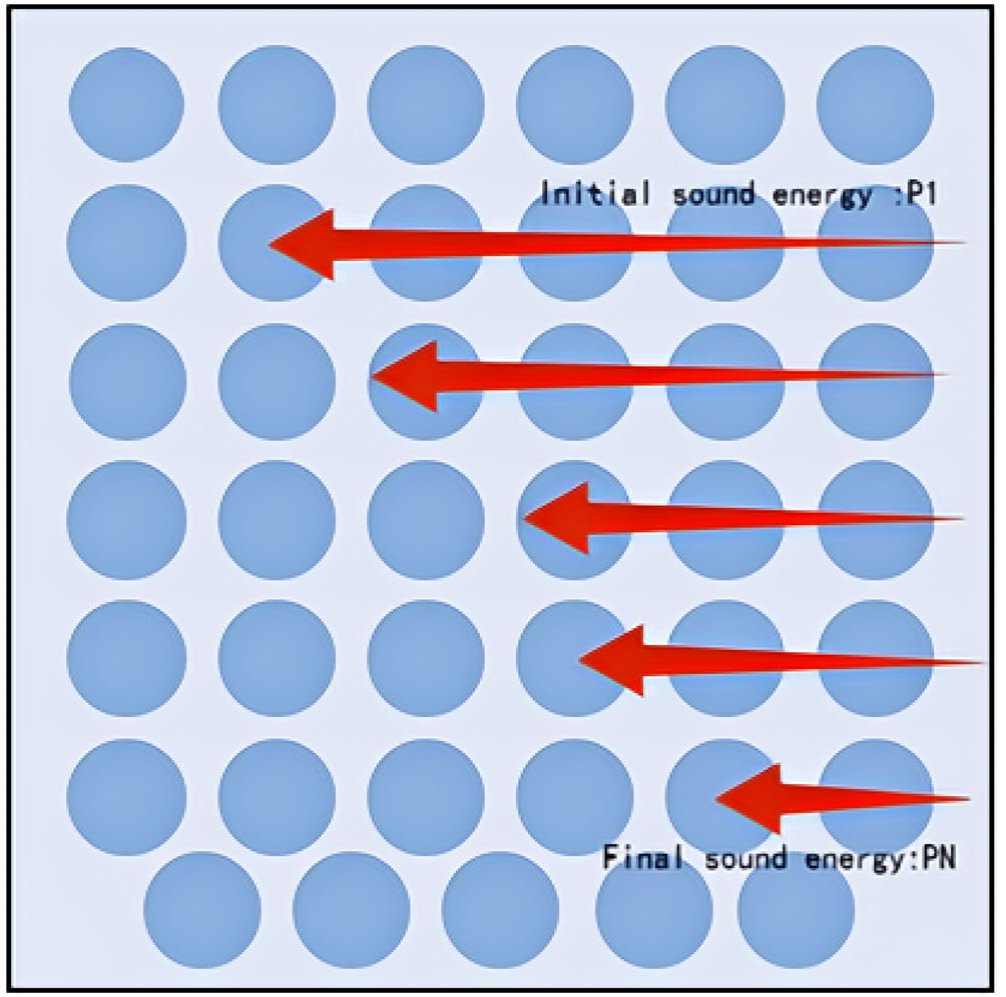
Acoustic energy is continuously weakened by friction in the “small ball" of the system.

**Figure 4:**
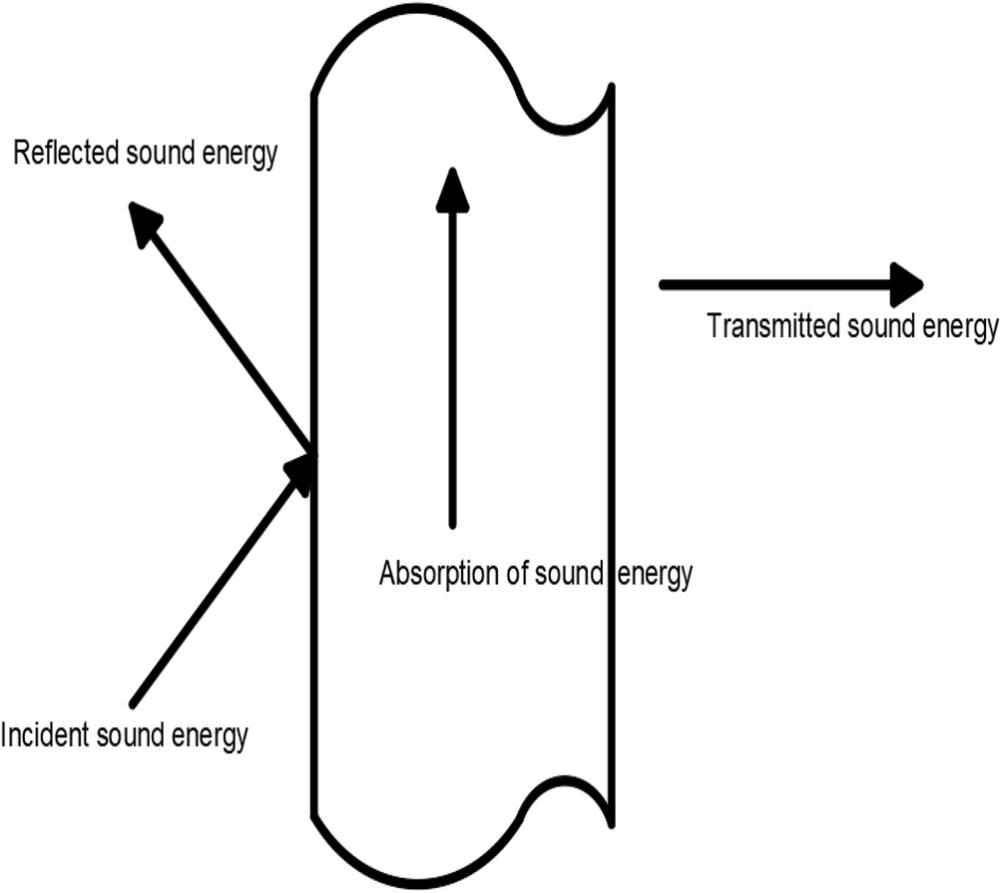
Diagram of sound absorption process of porous sphere foam system.

1. Porous ball foam system "small ball" sound insulation, sound absorption, heat preservation characteristics of the design: "small balls" can absorb sound not due to the surface roughness, but because of the large number of connections inside and outside the tiny pores and holes. When the sound wave is incident to the centrifugal glass wool, the sound wave can follow the pores into the material inside, causing the air molecules in the gap to vibrate. Due to the viscous resistance of the air and air molecules and the friction of the pore wall, acoustic energy is converted into thermal energy and loss. Secondly, due to the "small ball" of the porous viscosity and the role of internal friction, due to the propagation of sound waves, and the vibration speed of the mass in different places, there is a speed gradient. So that the interaction between neighbouring points of viscosity or internal friction, the mass of the movement of the point of impediment to the role of the acoustic energy is constantly converted into heat, thus playing a sound insulation, sound absorption, and heat preservation effect, as shown in Fig. 3.
2. Carbon fibre wave network inlay technology fixed "small ball": carbon fibre wave network inlay fixed with: (a) high strength, high modulus, lightweight; (b) Corrosion-resistant, Fatigue-resistant; (c) Construction is convenient, long service life; (d) It can be cut according to the need to adapt to the different reinforcing needs and other characteristics. Which divides a number of areas fixed to ensure that the system is laid on the surface of the floor slab under high strength pressure without falling off, and deforming. As well as the system laid in the outer wall enclosure to deal with the harsh environment of the wind and sun will not appear to have "resonance", which leads to the system being in the "small ball" off the situation.
3. "Carbon fibre acoustic panels" design ideas: the system "ball" around the "carbon fibre acoustic panels", cavity spacing "10–15 mm" equal distance distribution. The purpose of this is that porous acoustic materials can make a good sound energy absorption because of their viscous air resistance, fibre oscillation, and conflict, such as the transformation into thermal energy dissipation. So, it acts as a noise absorber. Carbon fibre sound-absorbing board has a good sound-absorbing effect on high-frequency noise, and the oscillating-type sound-absorbing material uses the force of acoustic pressure to make the board oscillate, which makes the internal consumption of acoustic energy play a role in the sound-absorbing characteristics, shown in Fig. 4.

### Design principles of sound insulation and thermal performance test methods and material performance analysis

**Figure 5:**
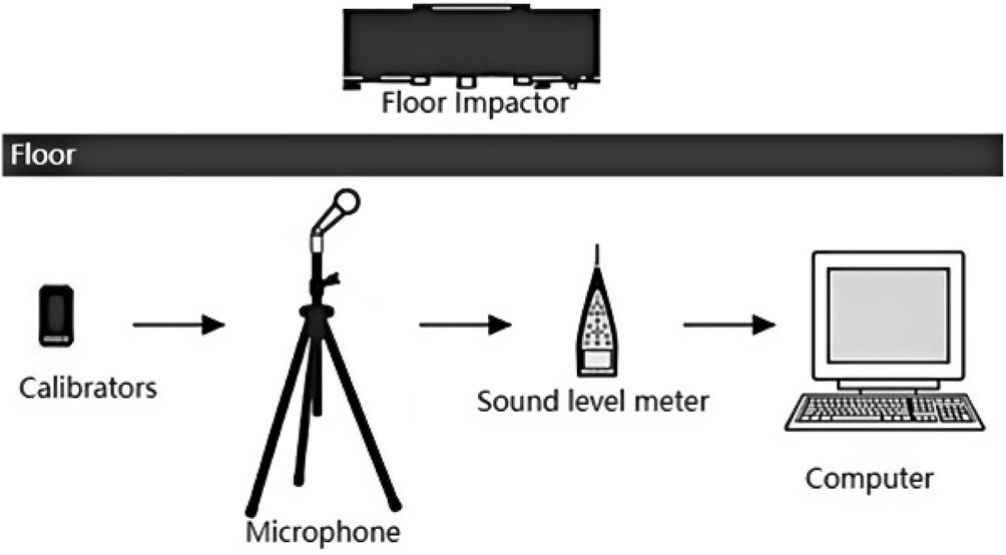
Floor impact sound insulation test equipment diagram^33^.

**Figure 6:**
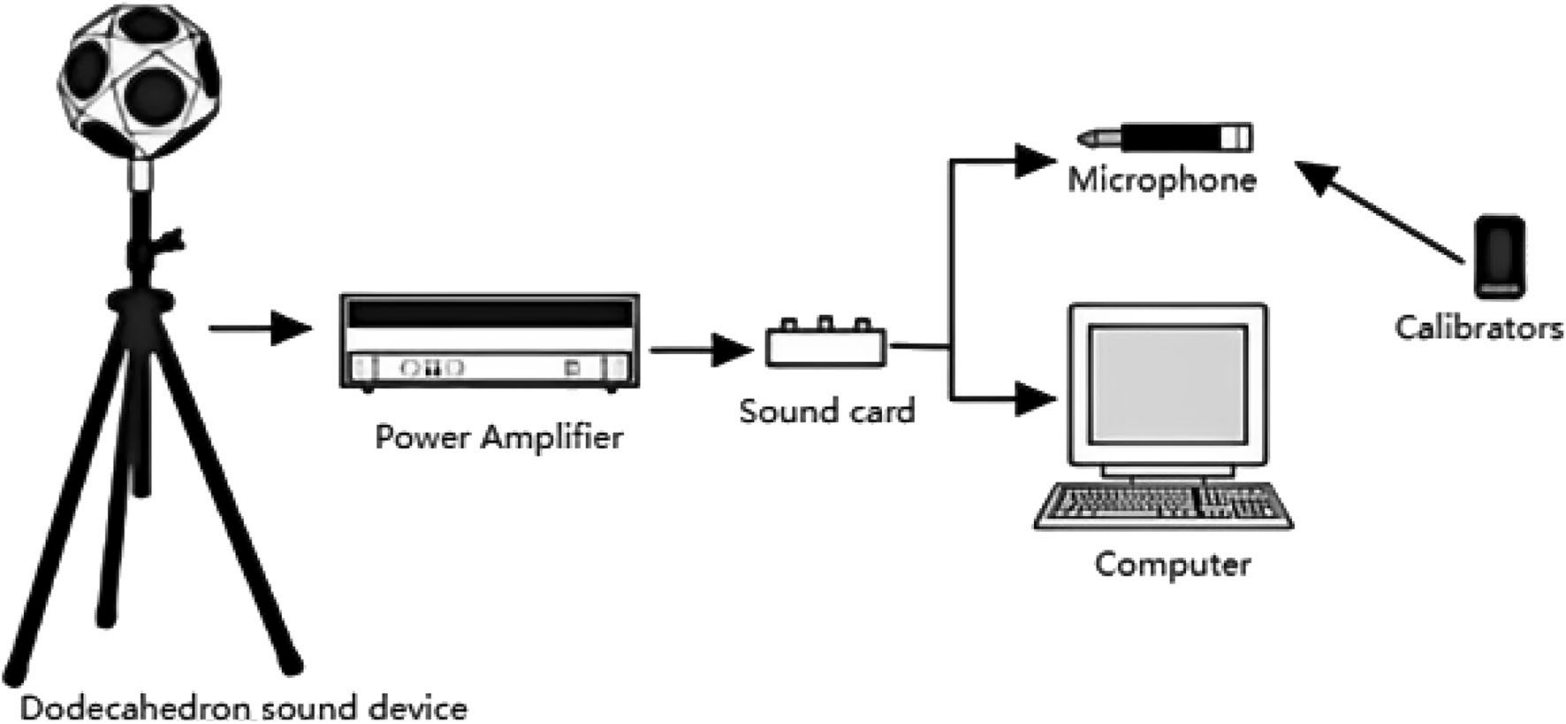
Floor and exterior wall airborne sound insulation test equipment diagram^33^.

The sub-study used is 1/3 octave, and the testing equipment is produced by NTI, a professional acoustic equipment manufacturer in Switzerland, which includes standard percussion, a sound level calibrator, a sound level meter, a dodecahedral sound source, a microphone, a power amplifier, and a multi-channel testing system, etc. As shown in Figs. 5 and 6. The selected test method is based on the current national standard "Evaluation Standard for Sound Insulation of Buildings" (GB/T 50121-2005)^21^, "Code for the Design of Sound Insulation of Civil Buildings" (GB50118-2010)^22^, "Acoustics Measurement of Acoustic Insulation of Buildings and Building Components Part 7: On-site Measurement of Impact Sound Insulation of Floor Slabs" (GB/T 19889.7-2005)^23^, "Green Building Indoor Environment Testing Technical Standards" (DGJ32/TJ194-2015)^24^, "Acoustics Building and Building Component Sound Insulation Measurement Part 5: On-site Measurement of Airborne Sound Insulation of Exterior Wall Components and Exterior Walls "(GB-T 19889.5-2006)^25^ as a backing. So, it has a stronger degree of acceptance in the country, the resulting data have a higher degree of scientific reliability, and the final analysis results are more accurate.

**Figure 7:**
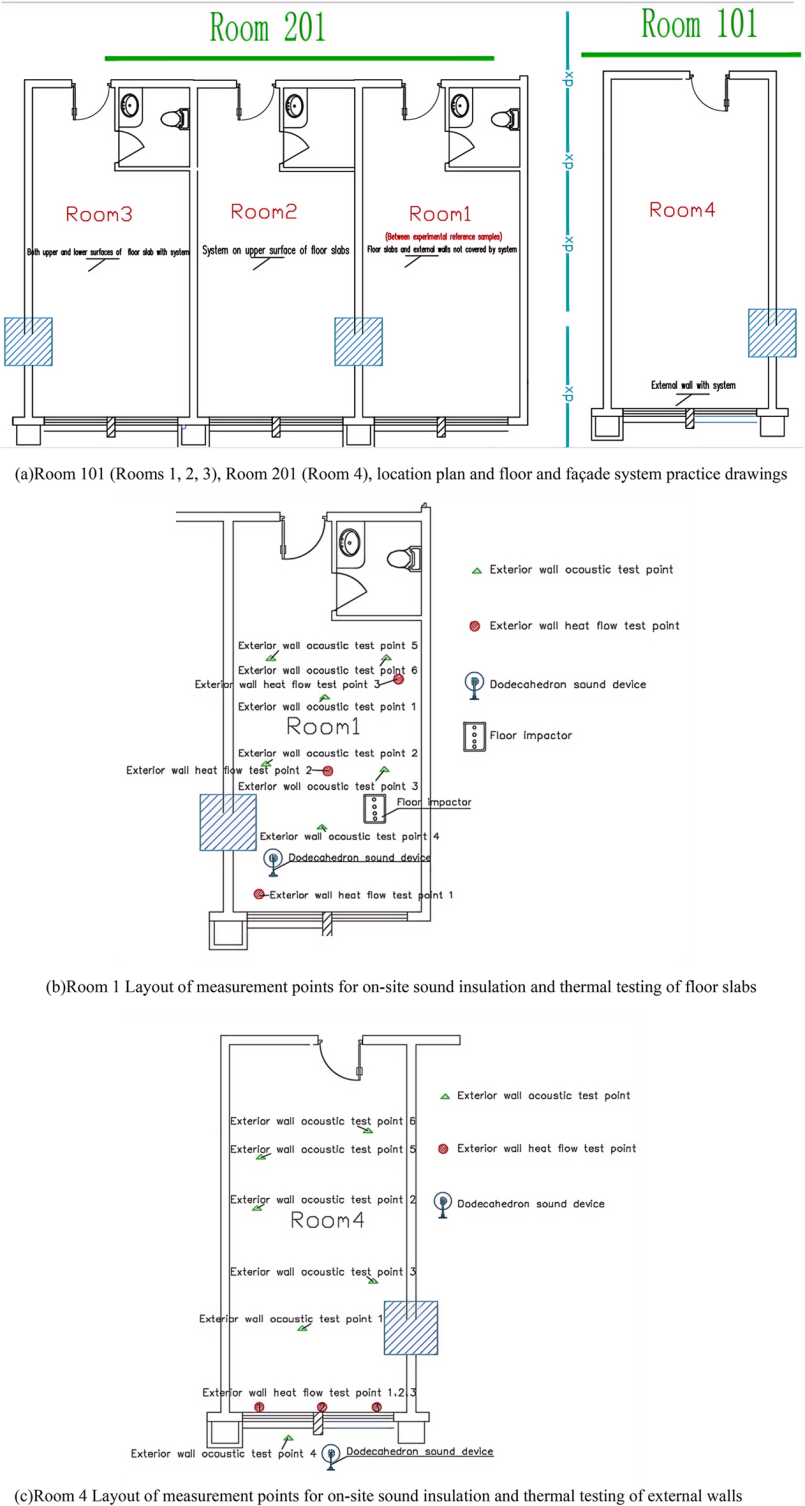
Arrangement of measurement points for sound insulation test and thermal test in some rooms.

In this study, the sound insulation performance of Room 101 and Room 201 (Room 1, Room 2, Room 3 sub-floor slab, and Room 4 exterior wall) of a green residential building in Zhenjiang City was tested on site. Figure 7a shows Room 201 (Rooms 1, 2, and 3), Room 101 (Room 4), a location map and a system approach map. The specific programme is as follows: Room 1 have floor slab and external wall without any system (as a sample room for comparison experiments). Room 2 have the upper surface of the floor slab with the carbon fibre porous sphere foam system. Room 3 have both upper and lower surfaces of the floor slab with the carbon fibre porous sphere foam system. And Room 4 have external wall with the carbon fibre porous sphere foam system. The tests points and equipment are shown in Fig. 7b,c. Here we illustrate that Room 101 (Room 4) is on the 1st floor, and Room 201 (Rooms 1, 2, 3) is on the 2nd floor, and their relationship is up and down, and such a design will facilitate the analysis of the results of the actual on-site comparative experiments that follow. Since the structure of Room 1 is the same as that of Room 2 and Room 3, the sound insulation and thermal testing of the floor slabs in Room 2 and Room 3 were carried out in the same manner as in Room 1. Refer to the test distribution in Fig. 7b.

**Figure 8:**
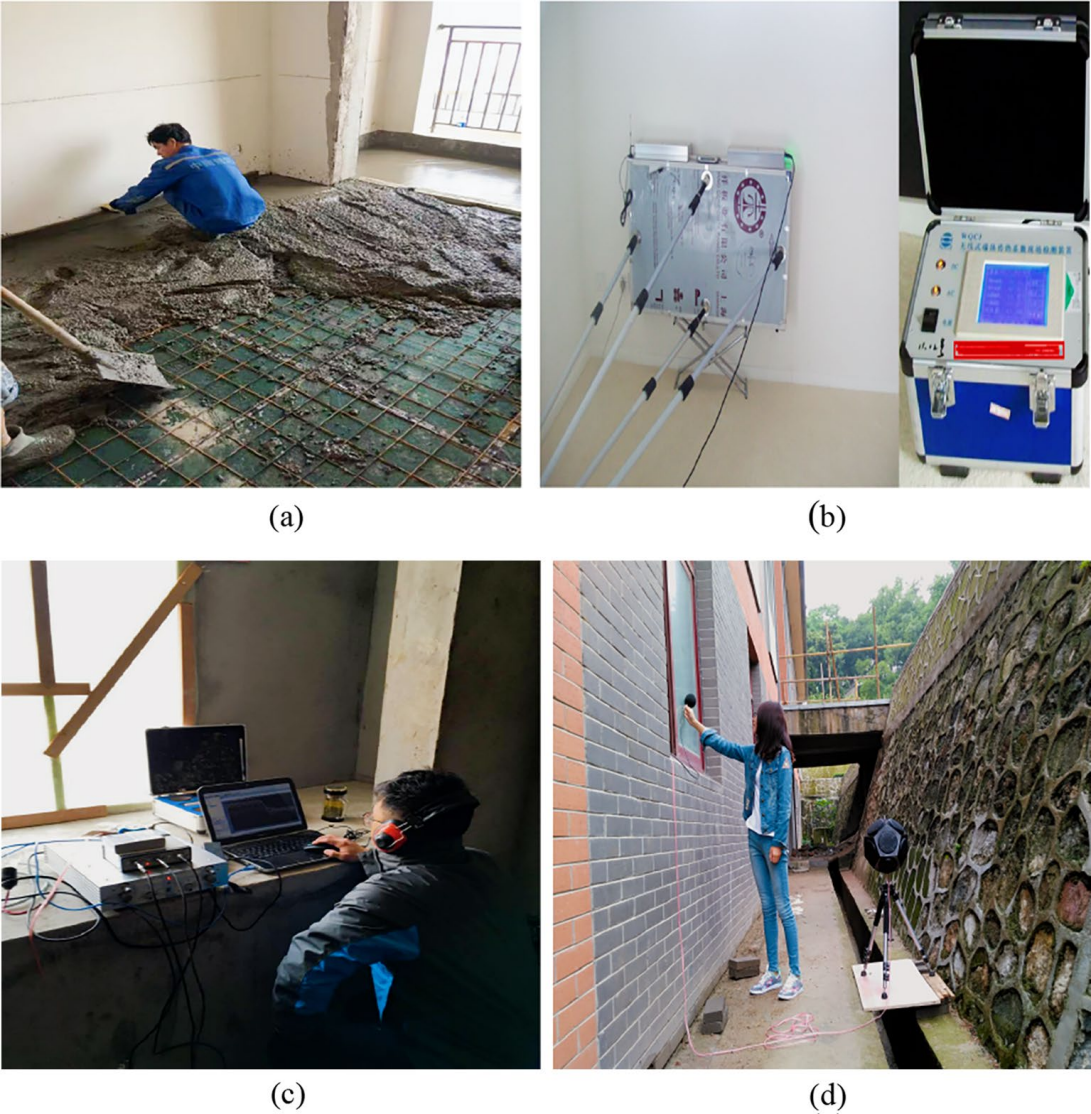
Photographs of material laying (**a**), thermal performance test (**b**), floor sound insulation test (**c**), external wall sound insulation test (**d**).

The thermal performance test adopts the thermal resistance tester of the enclosure structure (JSKS) according to the relevant provisions of the "On-site Thermal Performance Detection Standard for Civil Building Energy Conservation Engineering" (DGJ32/J23-2006)^26^. The heat flow meter method^27–32^ was used to carry out on-site tests on the heat transfer resistance of the floor slabs in Room 101(Room 4) and Room 201 (Room 1, Room 2, Room 3), Room 101 (Room 4) the external wall heat transfer resistance was tested on-site. Photographs of material laid on site prior to testing are shown in Fig. 8a.The test method is to place heating facilities in the test room to keep the room temperature near a fixed temperature, and use T-type thermocouples and heat flow meters to measure the temperature and heat flow. The test was conducted from 20 to 28 January 2023, and the test points and equipment are shown in Figs. 7c and 8b, and the photographs of the site of the above acoustic insulation and thermal testing are shown in Fig. 8b–d.

#### Principle of testing airborne sound insulation performance of floor slabs and external walls and analysis of material performance

1. Loudspeaker position

When measuring the sound insulation of floor members using loudspeaker noise, the distance r of the sound source from the centre of the member to be measured is at least 5 m (d > 3.5 m). When using loudspeaker noise measurement method to measure the external wall, r is at least 7 m (d > 5 m), and the angle of incidence of the sound wave should be 45 °C ± 5 °C^24,25^.

(2) Microphone position

Microphones are placed in the receiving room, and at least five microphone positions are used in each room to measure the average sound pressure level in each sound field. Where possible, the microphone positions should be no less than the following distances: (a) 0.7 m between microphones; (b) 0.5 m between any microphone position and a room interface or object; and 1.0 m between any microphone position and the test piece^24,25^.

Calculation of airborne sound insulation measurements for floor slabs and external walls:

1 $$ D_{nTw} = D + 10\lg \left( {\frac{T}{{T_{0} }}} \right) $$

For residential *T*_*0*_ = 0.5.

2 $$ D = L_{1} - L_{2} $$

Equation (2)* D* represents the sound pressure level difference between the average sound pressure level *L*_*1*_ from the sound source and the space- and time-averaged sound pressure level *L*_*2*_ in the receiving room. So when the reverberation time *T, T*_*0*_ is certain, the value of *D* difference becomes larger, and the value of *D*_*nT,w*_ increases, thus the airborne sound insulation performance of the floor and exterior wall components is enhanced. Because of the system of activated carbon fibers, wood chips, high temperature phenolic resin, high temperature glass beads, and ultrafine inorganic rock wool fibers, these materials have excellent acoustic properties. According to the above ratio foamed into a porous "small ball", on the outer surface of the external wall or the lower surface of the floor plate (and the receiving room is fully in contact with the surface), it will absorb some of the noise. This may improve the airborne sound insulation performance of floor slabs and external walls.

#### Principle of floor impact sound insulation performance testing and material performance analysis

The microphone shall measure the band sound pressure level at six measurement points distributed randomly in the receiving room. For each impinger position, there shall be a measurement point in the receiving room corresponding to the position of the impinger at a distance of 0.7 m or more from the boundary of the house. 1/3 octave band sound pressure levels shall be measured at each measurement point in the receiving room with the impinger in operation at 1/3 octave bands centred on the frequencies of 100 Hz, 125 Hz, 160 Hz, 200 Hz, 250 Hz, 315 Hz, 400 Hz 500 Hz, 630 Hz, 800 Hz, 1000 Hz, 1250 Hz, 1600 Hz, 2000 Hz, 2500 Hz and 3150 Hz. Standard impact sound pressure level to evaluate the impact sound insulation performance of the floor members, which is obtained by subtracting the correction term from the impact sound pressure level. The correction term is equal to the ratio of the reverberation time *T* in the receiving room to the reference reverberation time *T*_*0*_ in the common logarithm multiplied by 10. The standardised impact sound pressure level is expressed as *L'*_*nT,w*_*.*

3 $$ L^{\prime}_{{_{nTw} }} = L_{i} - 10\lg \frac{T}{{T_{0} }} $$

For residential homes, here *T*_*0*_ = 0.5 s^23^.

1. The carbon porous ball foam system designed in this paper has a certain amount of activated carbon fibre, wood chip powder, high temperature phenolic resin, high temperature glass beads, and ultrafine inorganic rock wool fibre. These materials have excellent acoustic absorption, in accordance with the above ratios foamed into a porous "ball". When the system is attached to the lower surface of the slab (and the receiving room contact), it can effectively reduce the receiving room reverberation time *T*, *T*_*0*_ is not the case, the standardised impact sound pressure level can be expressed as *L'*_*nT,w*_ will become smaller. So it can effectively reduce the reverberation time of the receiving room *T*, in the case of *T*_*0*_ does not become, by the formula (3) can be obtained, the standardised impact sound pressure level expressed as *L'*_*nT,w*_ will become smaller. So, the receiving room to receive the noise is also reduced, thus improving the impact sound insulation performance of the floor slab.
2. When the system is laid on the upper surface of the floor slab by the "floating" method, the floor slab, as a load-bearing member according to the requirements of structural strength, must have a certain thickness and surface density. According to the quality theorem of airborne sound insulation, there is a certain airborne sound insulation, such as that commonly used in residential 120 mm reinforced concrete plus renovation layer of airborne sound insulation in 48–50 dB. Plus, other construction measures, it can basically meet the airborne sound insulation requirements. As the concrete floor slab is more rigid, it is much more difficult to isolate the impact sound, and the interference problem in the lower room is more serious. For an infinite area, homogeneous solid, thicker floor slab, the impact sound pressure level *L*_*N*_ of the room under the floor slab excited by the impact is calculated as follows:
  4 $$ L_{N} = 20\lg \frac{{f^{1/4} }}{{E^{3/8} \times p^{5/8} \times h^{1/75} }} + {\text{Constant}} $$

where *E* is the modulus of elasticity of the floor slab (N/m^2^), *p* is the density of the floor slab (kg/m^3^), and *h* is the thickness of the floor slab (m). As can be seen from the formula, *L*_*N*_ increases with the increase in frequency and decreases with the increase in *E, p,* and* h*. Therefore, it can be achieved by increasing the *E, p*, and *h* of the floor slab. Therefore, the impact sound insulation capacity can be improved by increasing the *E, p*, and* h* of the floor slab, which is most effective for increasing the thickness of the floor slab. Every doubling of the thickness reduces the *L*_*N*_ by about 10 dB, but the increase in thickness is not economically practical, so other ways to solve the problem should be taken. The carbon porous ball foam system designed in this paper has a certain amount of high foaming elastic material. After the foaming process, when the system is laid on the upper surface of the floor slab, it will effectively increase the modulus of elasticity of the floor slab *E*, which effectively reduces the value of the impact sound pressure level *L*_*N*_ of the room under the floor slab. It has a certain effect on improving the sound insulation performance of the impact sound of the floor slab^33^.

#### Thermal performance testing principle and performance analysis of floor slabs and external walls

Calculation of average temperature and heat flow time-by-time value: The time-by-time value of the average temperature of the inner and outer surfaces of the enclosure shall be calculated according to the following formula:

5 $$ \theta_{i,i} = \sum {\theta_{i,ij} \times A_{j} } /A $$

6 $$ \theta_{e,i} = \sum {\theta_{e,ij} \times A_{j} } /A $$

$\theta_{i,i}$,$\theta_{e,i}$ Represents the hourly value of the average temperature of the inner and outer surfaces of the enclosure (°C). *A*_*j*_ Area represented by the jth sensor (m^2^). *A*—Component area (m^2^).

The time-by-time value of heat flow through the enclosure is calculated by the following formula:

7 $$ Q_{i} = \sum {Q_{ij} \times A_{j} } /A $$

The data were analysed using the arithmetic mean method, and the measured value of heat transfer resistance was calculated using the formula:

8 $$ R = (\theta_{i,i} - \theta_{e,i} )/Q_{i} $$

The test data of the first 4*d* and the last 4*d* of the thermostatic process were used for the calculation, respectively, and the test data of the last 4*d* of the thermostatic process were used for the calculation of the R value when the difference in the calculated *R* value was not more than 5%. When exact data are available, the effect of the moisture content of the envelope material can be considered, and the thermal resistance can be corrected^26^.

**Figure 9:**
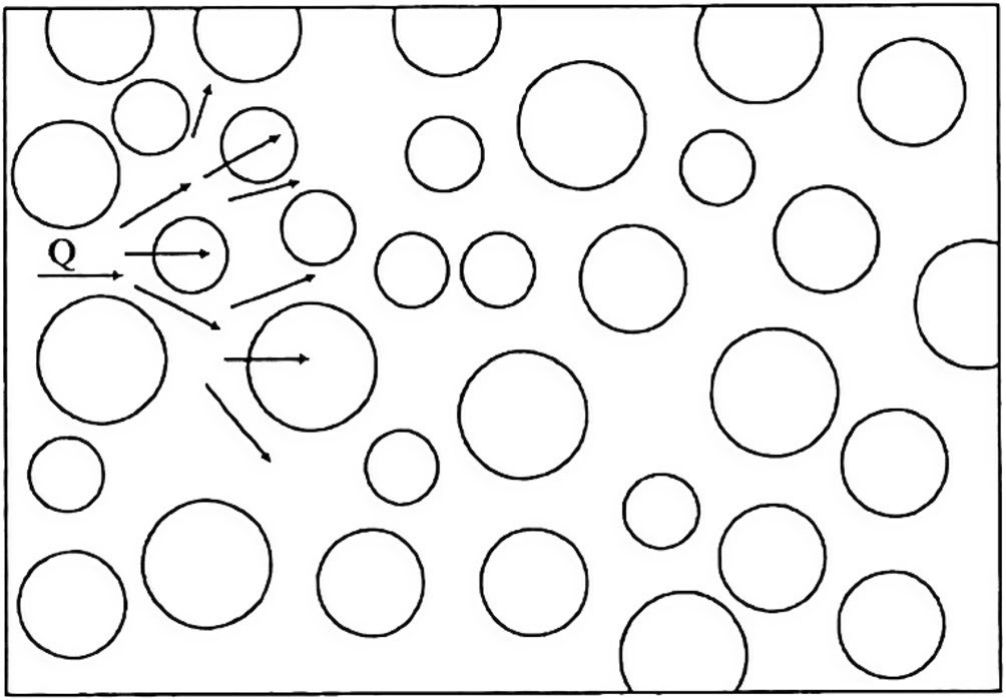
The process of heat conduction in a hole.

The carbon porous ball foaming system designed in this paper is made of porous material; the base material and the foam "ball" have a large number of closed pores. The heat transfer process in the pores, as shown in Fig. 9, the heat propagation in the pores, is divided into solid-phase heat conduction and heat transfer in the gas phase. In the solid phase, due to the presence of a large number of pores, the path of heat transfer changes, the total distance increases, and the heat transfer efficiency decreases. Heat transfer in the gas phase to the air thermal conductivity is dominated by the thermal conductivity of air, due to the thermal conductivity of the air is only 0.029 W/(m k), it much smaller than the solid-phase thermal conductivity. So, the transfer rate will become slower. In summary, the system has a certain degree of thermal insulation performance.

### Analysis of test results

#### Sound insulation performance and thermal performance of floor slabs and external walls when there is no systematic laying structure in the room

**Figure 10:**
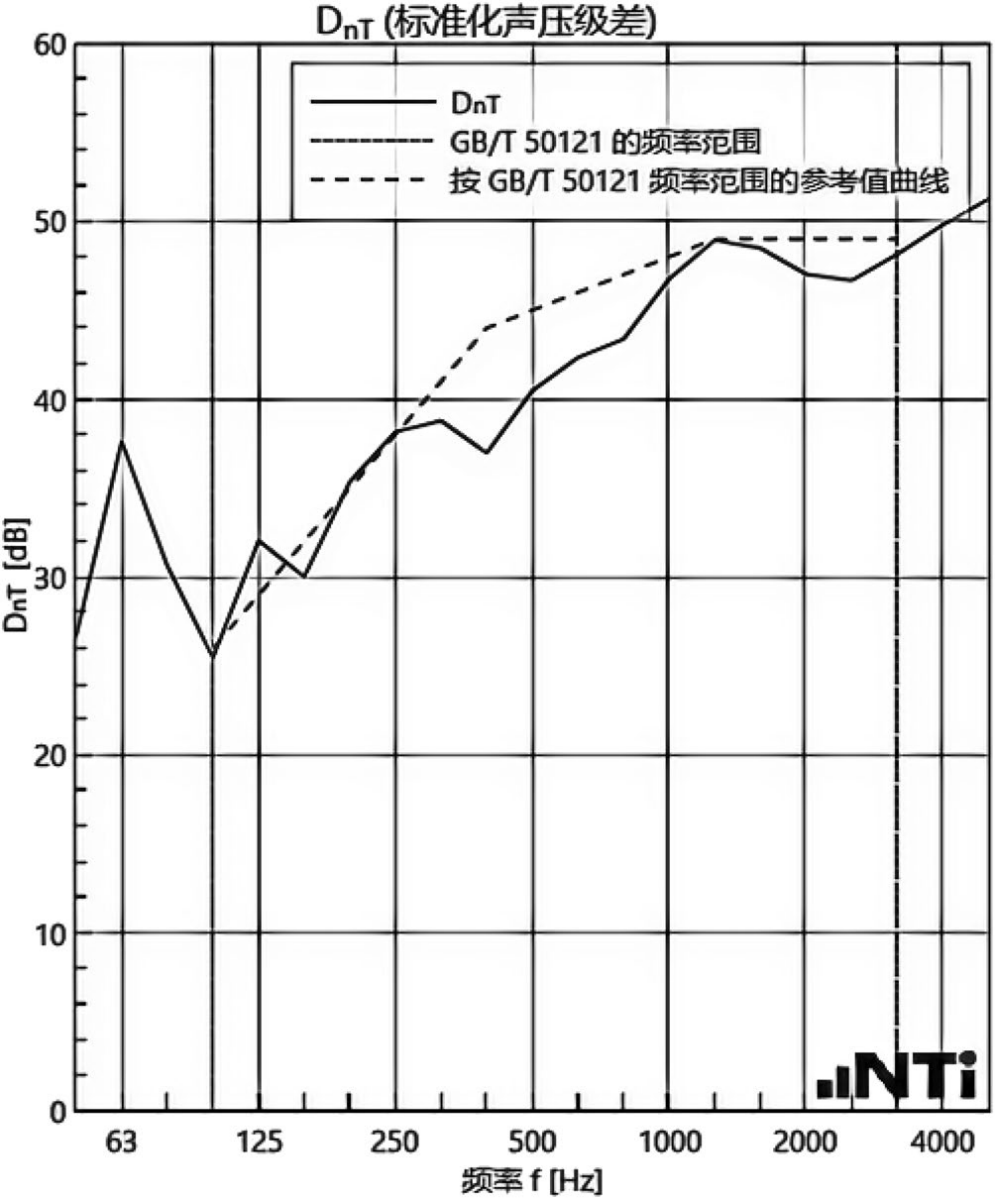
Airborne sound insulation performance curve for floor slabs of unsystematically laid structure.

**Figure 11:**
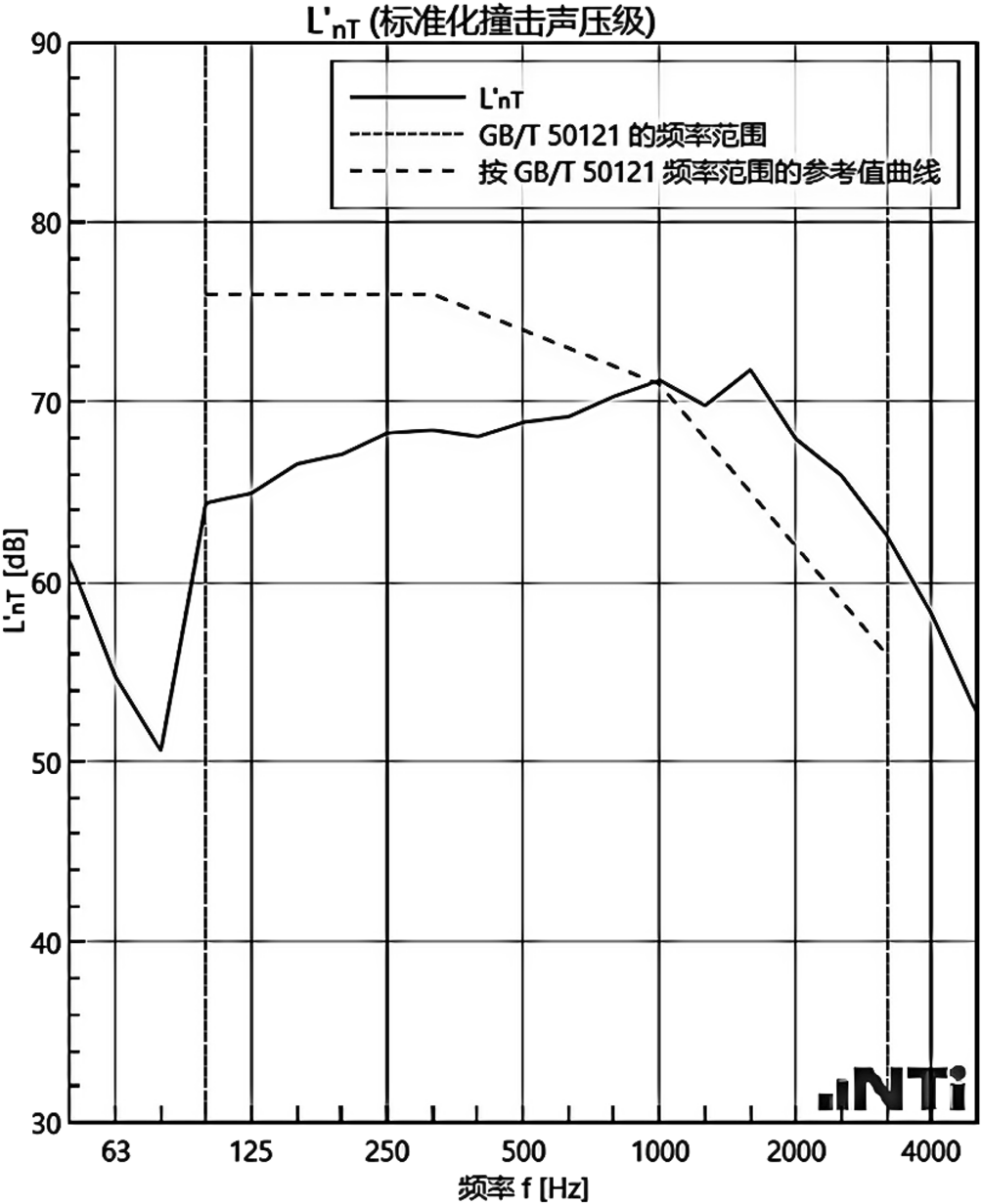
Impact sound insulation performance curves for floor slabs of unsystematically laid structure.

**Figure 12:**
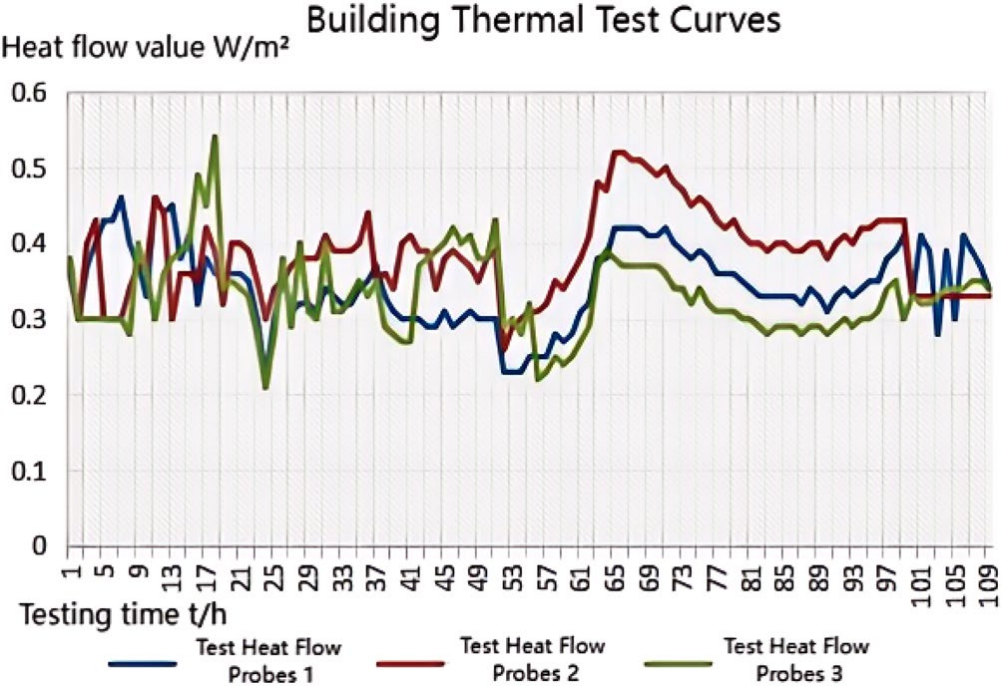
Heat flow test curves for thermal performance of floor slabs without system laying structure.

We experimented in Room 201 (room 1) sub-floor slabs on-site floor airborne sound, impact sound insulation. According to (GB/T 50121-2005)^21^ grading index, the experimental results are: weighted standard sound pressure level difference and spectral modification: *D*_*nT,w*_ + *C* = 45 dB − 0 dB, acoustic performance classification for the 7th level, the sound insulation curve. The sound insulation curve is shown in Fig. 10; the weighted standardised impact sound pressure level *L'*_*nT,w*_ = 74 dB, the sound insulation performance is graded as grade 2, and the sound insulation curve is shown in Fig. 11. These acoustic graphs are automatically generated by the NTI acoustic test system. The acoustic test results given in this paper, such as D_nT,w_ + C and L′_nT,w_, are taken at 500 Hz on the X-axis of the test curve and at the corresponding dotted line on the Y-axis according to the standard (GB/T 50121-2005)^21^.The test results of the floor slab thermal resistance *R*_*L1*_ = 2.37 m^2^ K/W, heat flow test curve are shown in Fig. 12. Three curves represent: real-time test data curves of 3 measurement points of the heat flow test.

**Figure 13:**
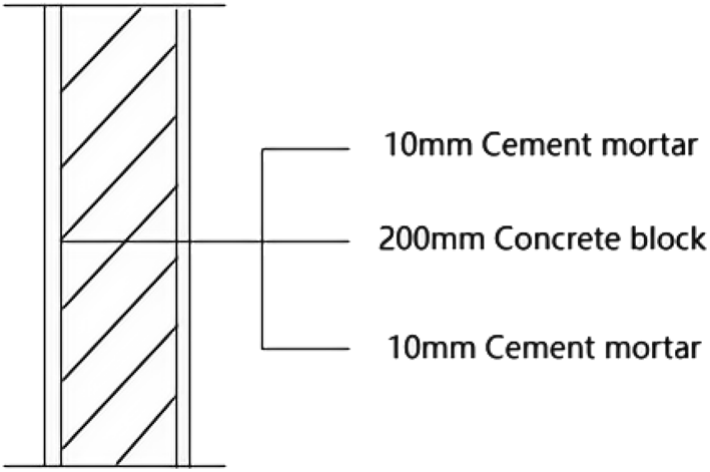
Exterior wall design practices in the absence of systematically laid structure.

**Figure 14:**
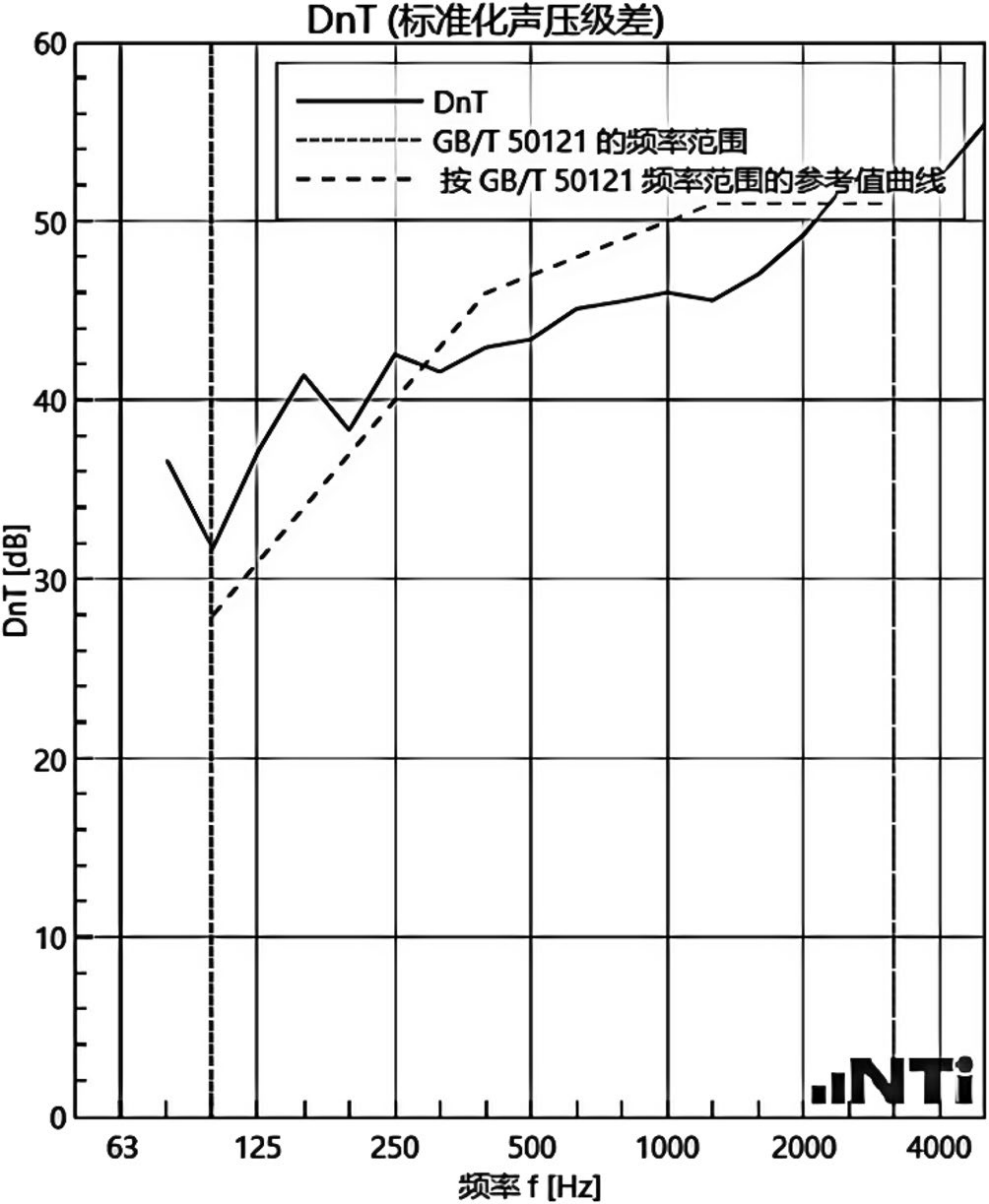
Performance curve of airborne sound insulation of exterior walls without systematic laying of structure.

**Figure 15:**
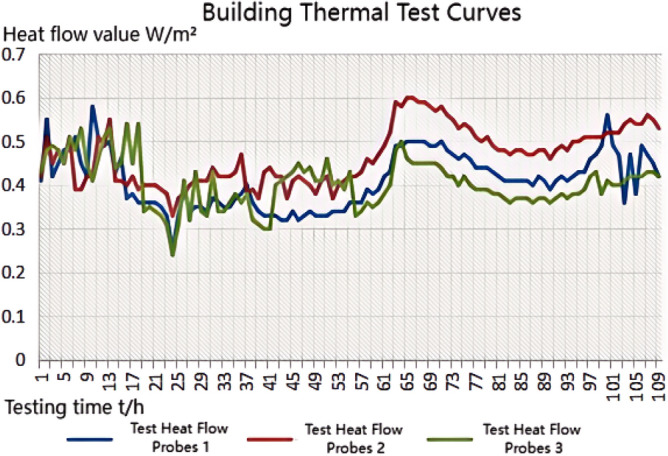
Heat flow test curves for thermal performance of external walls without system laying.

Room 201 (room 1) external wall specific layering practices are: 10 mm cement mortar + 200 mm aerated concrete thermal insulation external wall blocks + 10 mm cement mortar as shown in Fig. 13. In Room 1 external wall on-site external wall air sound insulation performance experiments, the experimental results are: The weighted standard sound pressure level difference and spectral correction are: *D*_*nT,w*_ + *C*_*tr*_ = 46 dB − 2 dB, and the sound insulation performance classification is 6 levels. The sound insulation curve is shown in Fig. 14. The test results of the thermal resistance of the external wall *R*_*Q1*_ = 1.99m^2^ K/W. The heat flow test curve is shown in Fig. 15.

#### Sound insulation and thermal performance of structures laid on the upper surface of the floor slab in room 2

**Figure 16:**
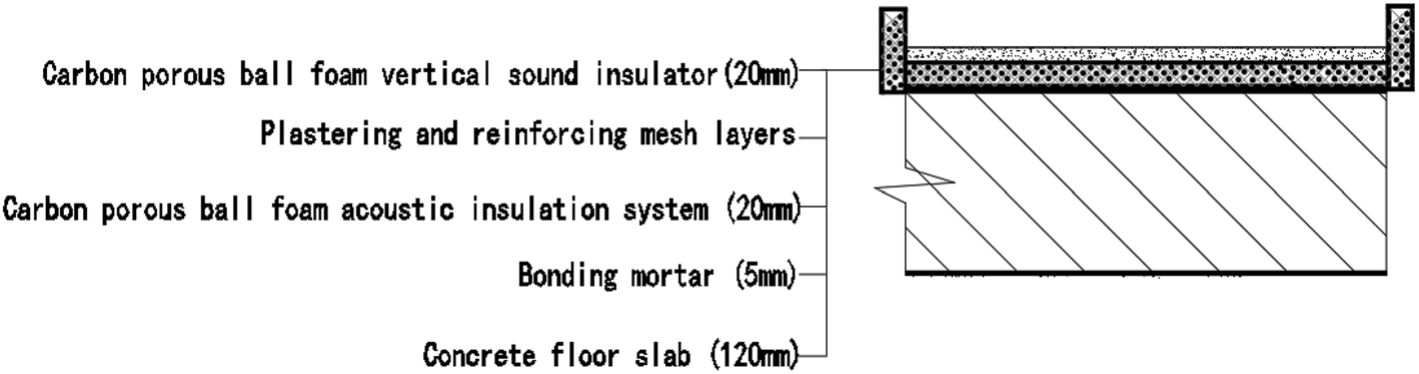
Design practice for structural floor slabs with upper surfaces.

**Figure 17:**
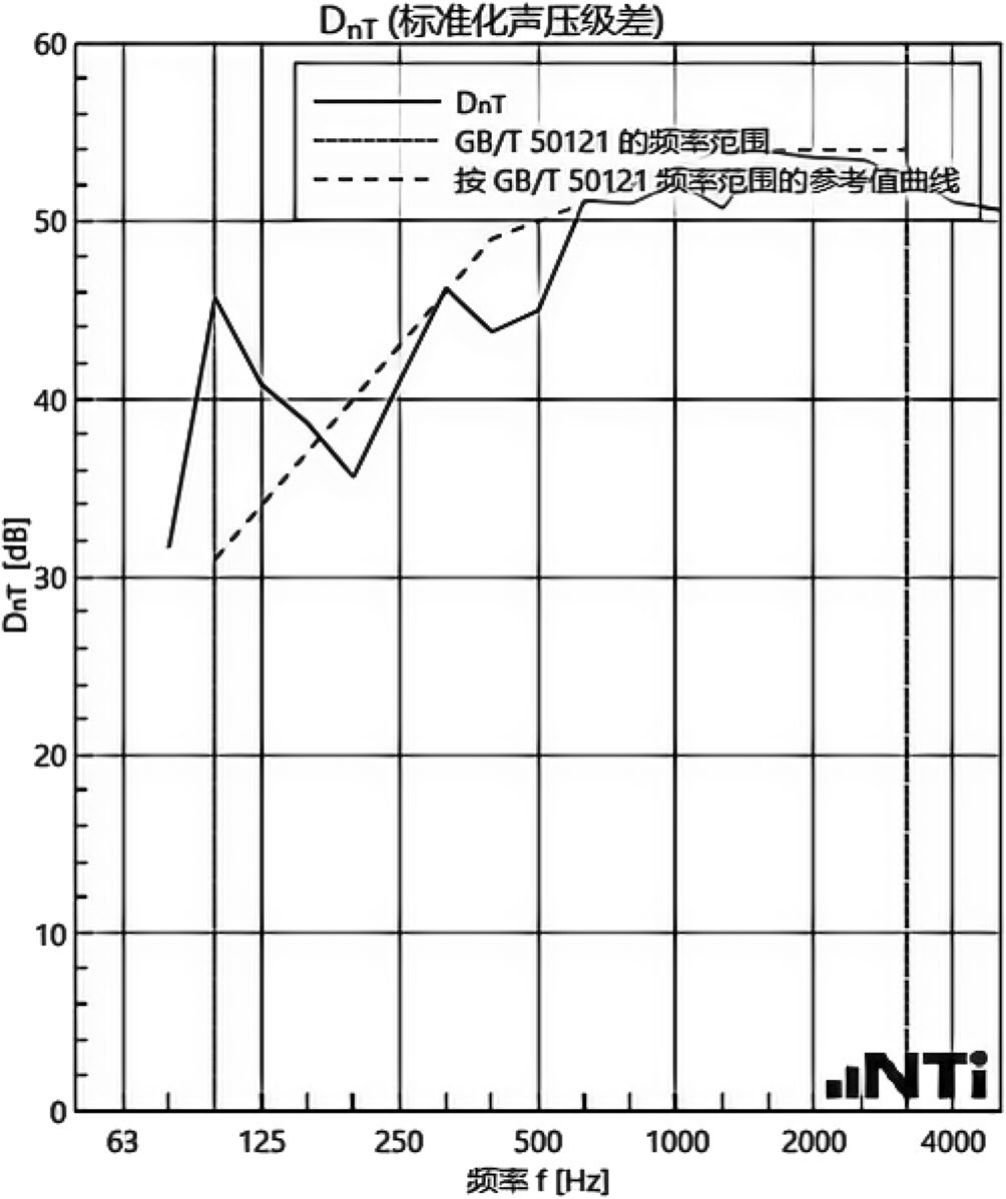
Airborne sound insulation performance curve for upper surface layup structural floor slabs.

**Figure 18:**
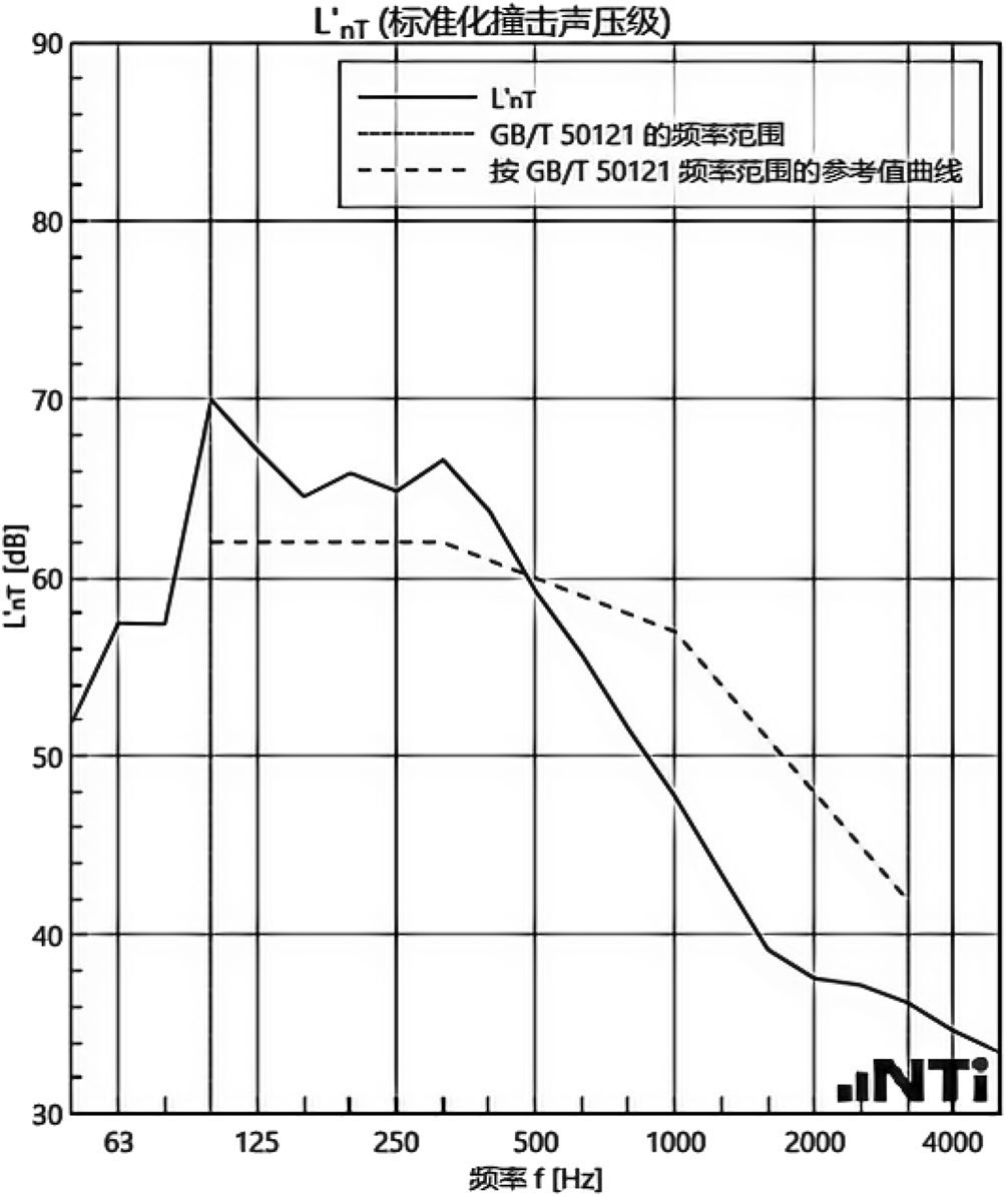
Impact sound insulation performance curve for upper surface laying structural floor slabs.

**Figure 19:**
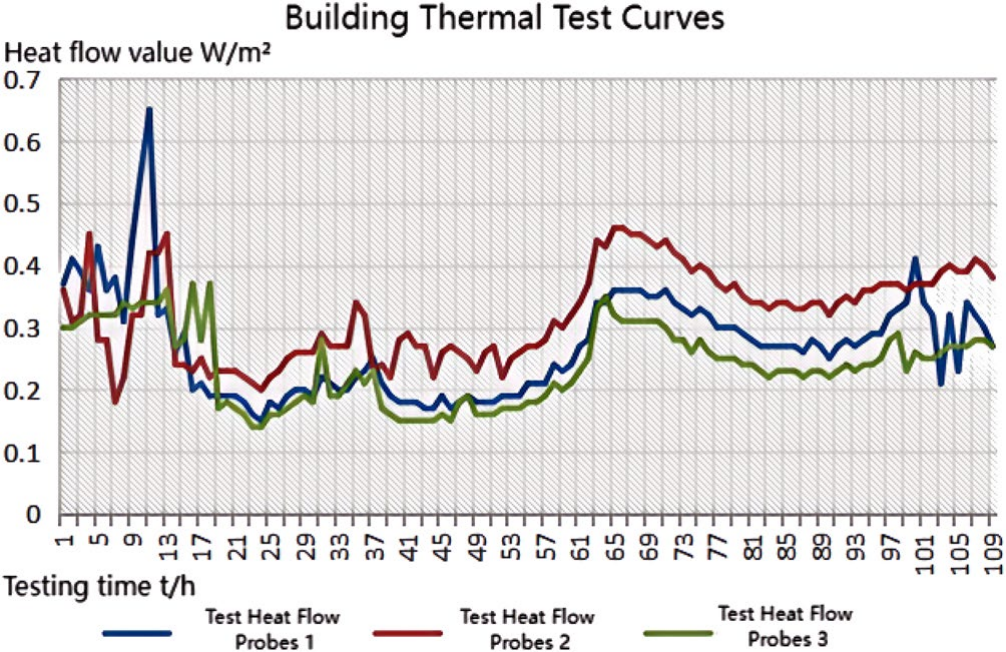
Thermal performance heat flow test curve for upper surface layup structural floor slabs.

Room 201 (room 2) sub-floor construction unit in the upper surface of the floor slab laid 20 mm sound insulation, thermal insulation carbon porous ball foam system, the specific layering practices are: floor for 10 mm plastered reinforced layer + 20 mm carbon porous ball foam system + 20 mm vertical acoustic pads (carbon porous ball foam system) + 120 mm cast-in-situ reinforced concrete floor slabs, layering design practices are shown in Fig. 16. From the floor slab of the field layering practices, in full compliance with the "Residential building floating floor insulation sound insulation engineering technical regulations" (DB32/T3921-2020)^34^. In the floating floor sound insulation material laying methods, and in the sound insulation board and the four walls between the vertical acoustic isolation pads, phase isolation is used to avoid the resonance of the interference. The author for the room 2 sub-floor slabs on-site floor airborne sound and impact sound insulation experiments, according to (GB/T 50121-2005)^21^ grading index experimental results are: weighted standard sound pressure level difference and spectral correction for *D*_*nT,w*_ + *C* = 50 dB − 3 dB acoustic performance is graded at level 7, sound insulation curve in Fig. 17; Weighted standardised impact sound pressure level *L*′_*nT,w*_ = 60 dB, sound insulation performance is graded as level 5, and the sound insulation curve is shown in Fig. 18. The test results of the thermal resistance of the floor slab, *R*_*L2*_ = 2.99 m^2^ K/W, are shown in the heat flow test curve in Fig. 19.

#### Room 3 floor slab Sound insulation performance and thermal performance of structures laid on the upper and lower surfaces of floor slabs

**Figure 20:**
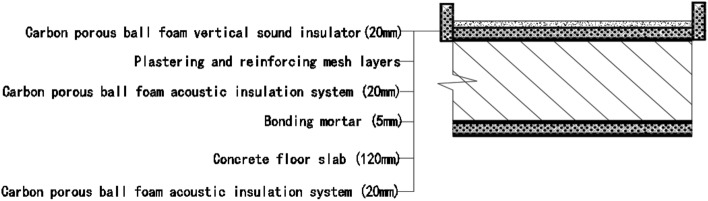
Design practices for structural floor slabs with upper and lower surface coverings.

Room 201 (room 3) sub-floor construction unit in the floor slab on the upper and lower surfaces laid 20 mm sound insulation, thermal insulation carbon porous ball foam system, specific layering practices are: floor for 10 mm smeared surface reinforcement layer + 20 mm carbon porous ball foam system + 20 mm vertical acoustic pads (carbon porous ball foam system) + 120 mm cast-in-situ reinforced concrete floor slab + 20 mm carbon porous ball foam system (lower to the type), layered design practice is shown in Fig. 20. The design of the lower surface laying is important because according to the carbon fibre porous ball’s high acoustic performance, it will change the floor plate’s air sound insulation performance, as elaborated in the above acoustic principle analysis.

**Figure 21:**
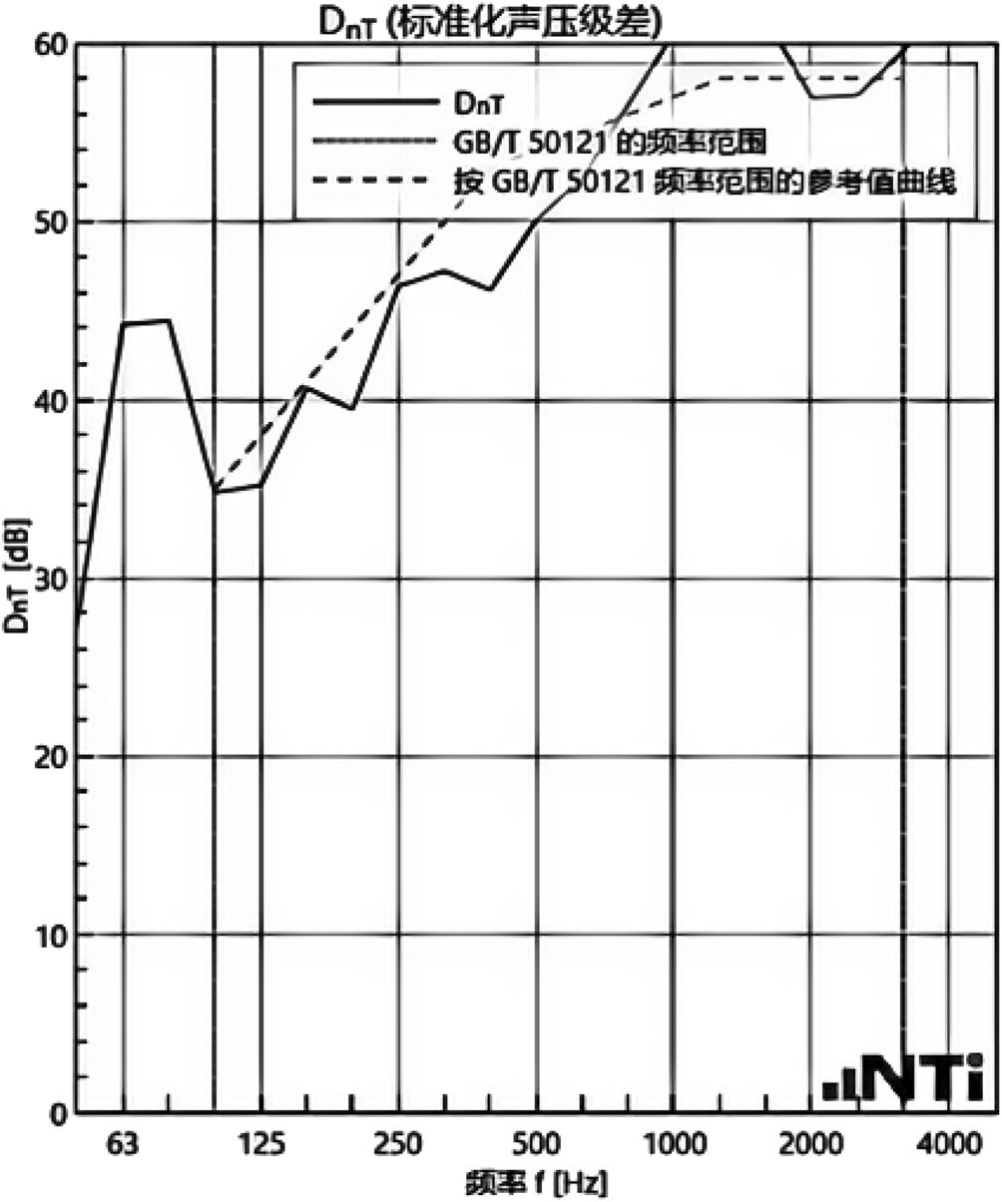
Airborne sound insulation performance curves for upper and lower surface laying structural floor slabs.

**Figure 22:**
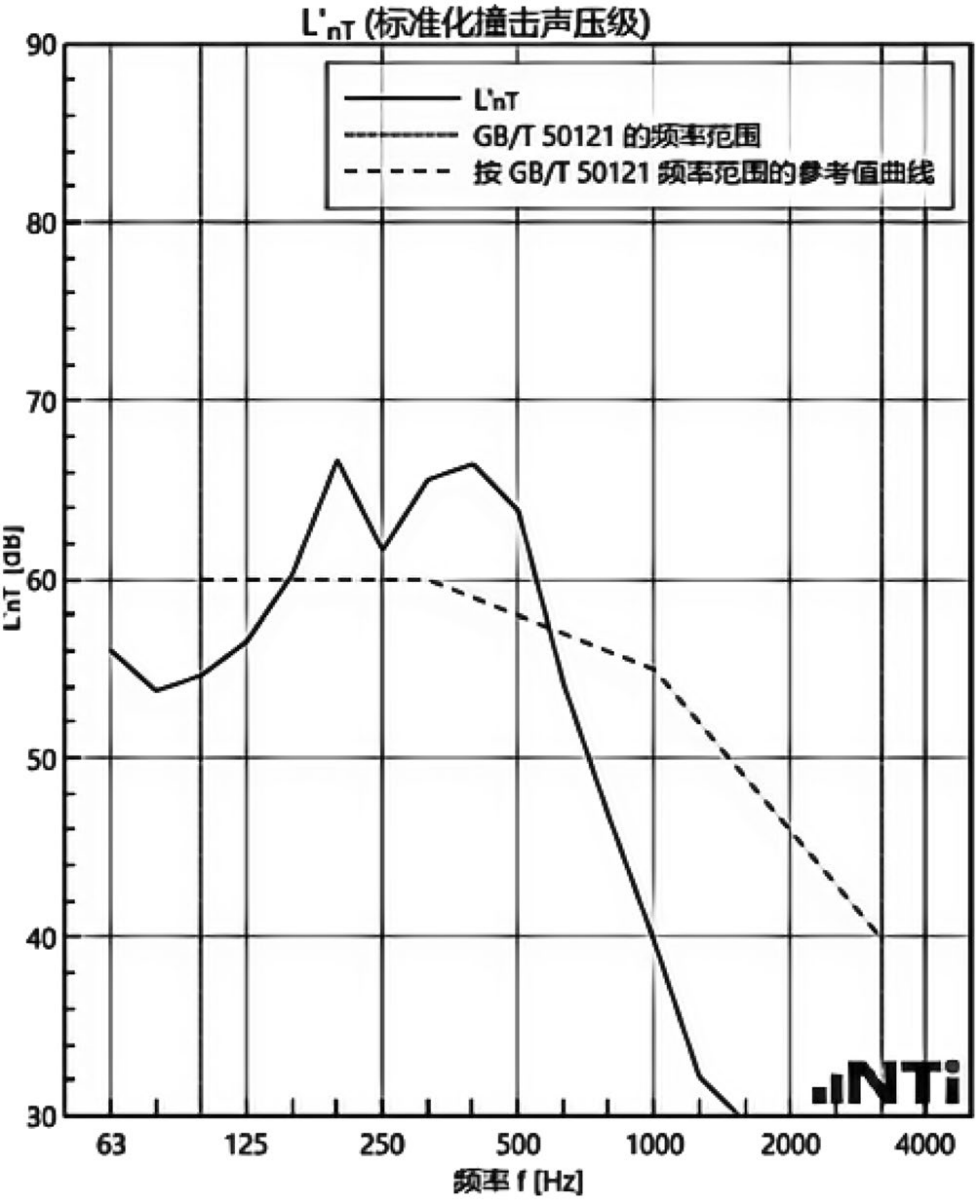
Impact sound insulation performance curves for upper and lower surface laying structural floor slabs.

**Figure 23:**
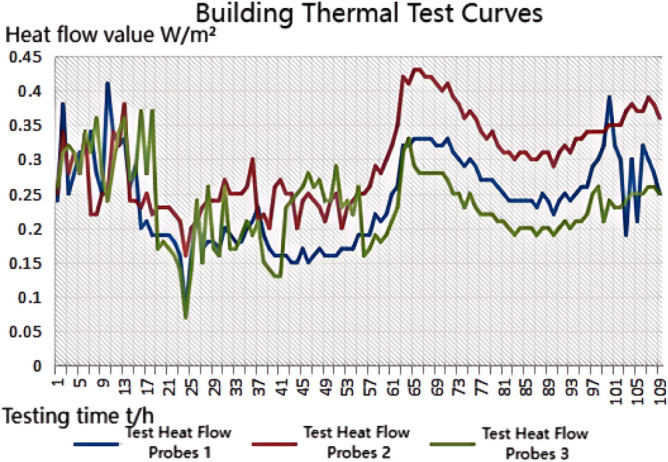
Thermal performance heat flow test curves for upper and lower surface laying structural floor slabs.

In room 3, the author sub-divided floor slabs for on-site floor airborne sound and impact sound insulation experiments. According to (GB/T 50121-2005)^21^ grading index experimental results are: weighted standard sound pressure level difference with the spectral correction: *D*_*nT,w*_ + *C* = 54 dB − 4 dB, sound insulation performance is graded at level 8, sound isolation curves are shown in Fig. 21; Weighted standardised impact sound pressure level *L*′_*nT,w*_ = 58 dB, sound insulation performance is graded as level 5, the sound insulation curve is shown in Fig. 22. The test results of the thermal resistance of the floor slab, *R*_*L3*_ = 3.41 m^2^ K/W, are shown in the heat flow test curve in Fig. 23.

#### Room 4 sound insulation and thermal performance of the external wall covering structure

**Figure 24:**
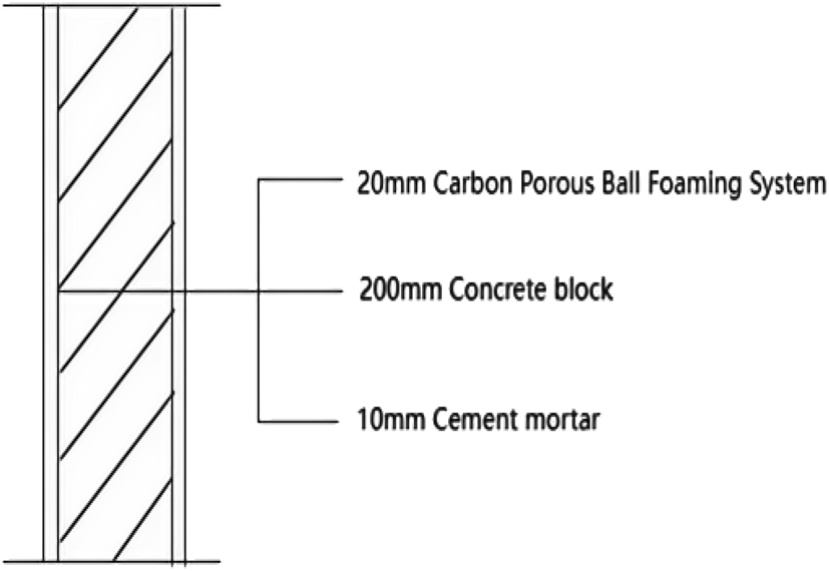
Design practices for structural floor slabs on exterior wall surfaces.

Room 101 (room 4) exterior wall construction unit laid 20 mm sound insulation, thermal insulation carbon porous ball foam system on the outside surface. Specific layering practices are: floor slab for 10 mm cement mortar + 200 mm aerated concrete thermal insulation exterior wall blocks + 20 mm carbon porous ball foam system. Layering design practices are shown in Fig. 24. Design of the lower surface paving because, according to the carbon fibre porous ball has a high acoustic and thermal resistance performance. The airborne sound insulation performance, thermal resistance performance of the external wall will be changed in the above acoustic principle analysis, which has been elaborated.

**Figure 25:**
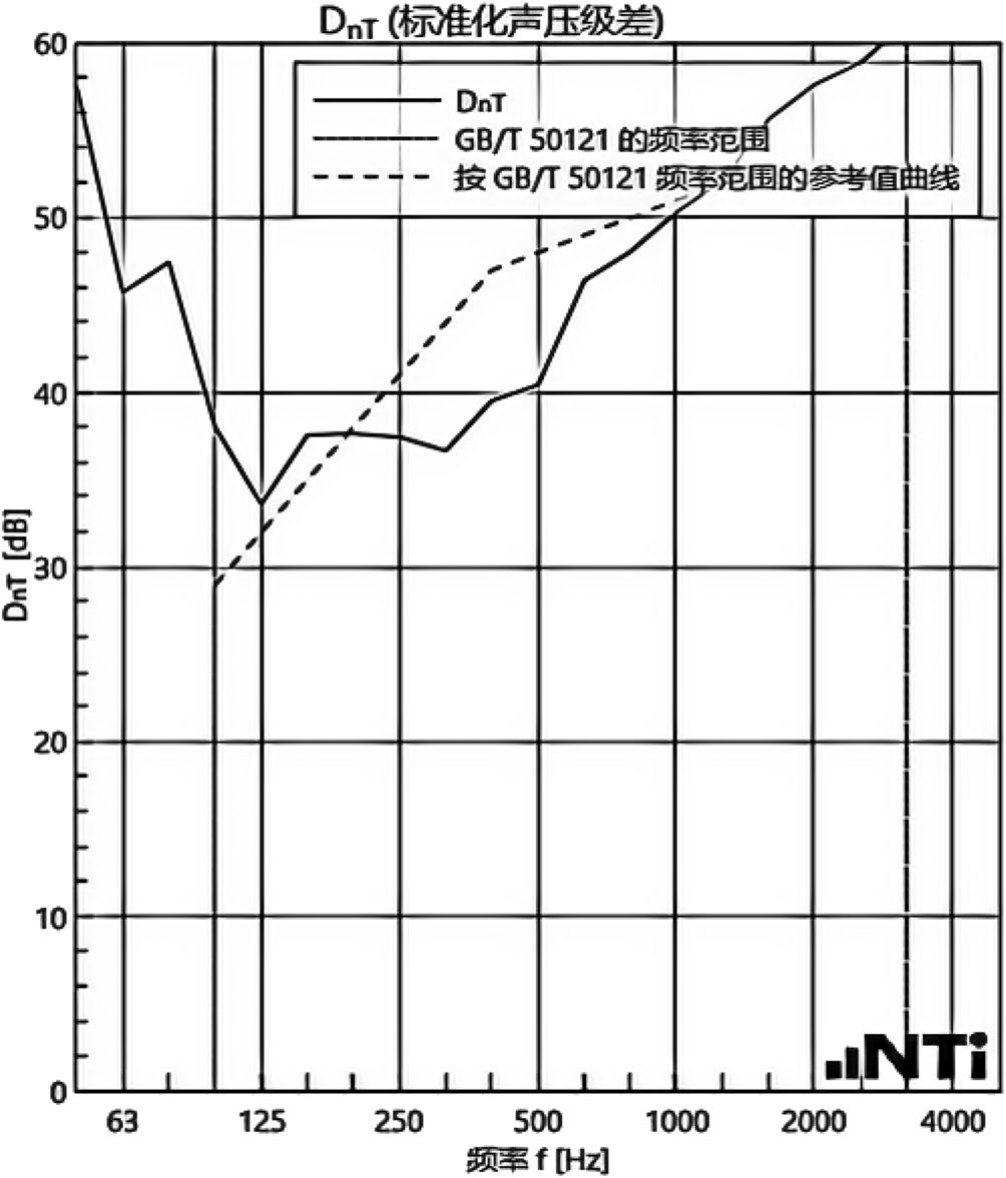
Performance curve of airborne sound insulation of external walls when laying the system.

**Figure 26:**
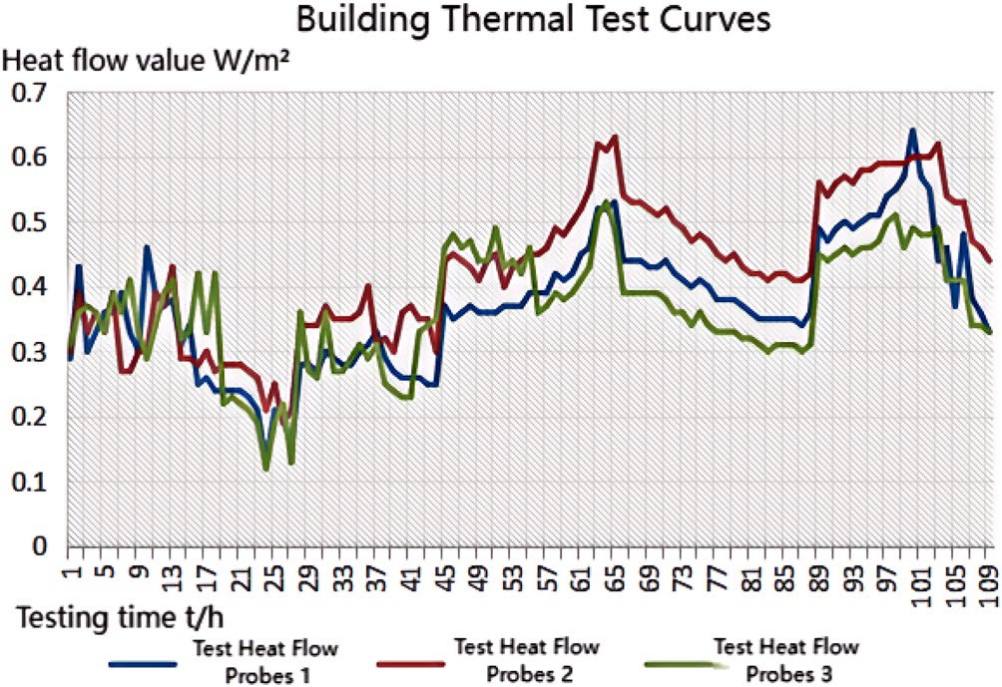
Heat flow test curves for thermal performance of external walls when laying the system.

In room 4 exterior wall on-site exterior wall airborne sound insulation performance experiments, according to (GB/T 50121-2005)^21^ grading index experimental results are: weighted standard sound pressure level difference and spectral correction for: *D*_*nT,w*_ + *C*_*t*r_ = 48 dB − 1 dB, sound insulation performance is graded at level 7, and the sound insulation curve is shown in Fig. 25. The test results of the floor slab thermal resistance *R*_*Q4*_ = 2.19 m^2^ K/W, heat flow test curve are shown in Fig. 26.

## Results and analyses

### Analysis of the results of sound insulation performance of floor slabs and external walls

**Figure 27:**
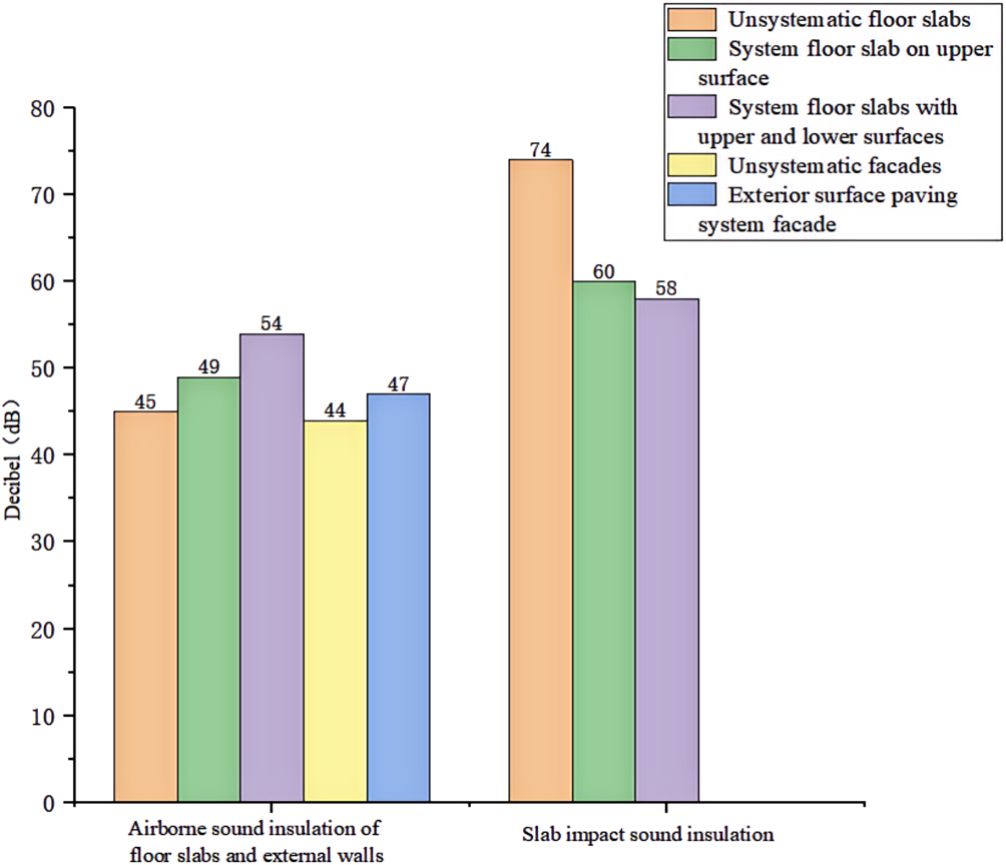
Comparison of results of sound insulation performance of floor slabs and external walls.

1. An Illustration in Fig. 27 shows the floor and wall acoustic performance results of the comparative histogram along with the floor without layer system for comparison: when the upper surface of the floor is paved with 20 mm sound insulation, thermal insulation, and a carbon porous ball foam system. As well as the use of the material system as a floor acoustic insulation system of vertical acoustic pads (to prevent the occurrence of resonance between the floor and the wall), the floor impact acoustic performance reduction from 74 dB (level 2) to 60 dB (level 5) can meet the current standard "Code for Sound Insulation Design of Civil Buildings" (GB50118-2010)^22^ high limit (floor impact sound insulation ≤ 65 dB) requirements. Effective floor impact sound enhancement of 14 dB is attributed to the acoustic insulation, heat preservation carbon porous sphere foam system of high temperature phenolic resins, high-foam elastic materials, such as the modulus of elasticity (Pa) of better materials, making the system made of foaming, and the system of foam (Pa).

Materials: making the system foam with a good modulus of elasticity (Pa), good flexibility, and damping characteristics, the vibration of the floor impact sound energy has a good attenuation effect, so that the impact sound insulation performance of the floor has been significantly improved. In addition to the skirting line after the detailed treatment (the vertical acoustic isolation piece of the blocking), block the reinforced concrete floor slabs and the walls, beams, and columns between the acoustic bridges, so that the floor's impact sound insulation performance has been significantly improved. When 20 mm of the system is laid on the upper and lower surfaces of the floor slab, the impact sound insulation performance of the floor slab is 58 dB (level 5). Although it is 16 dB higher than that of the unpaved system, it is not much higher than that of the upper surface of the floor slab, which means that the system laid on the lower surface of the floor slab does not have a significant effect on the improvement of the impact sound insulation performance of the floor slab.

(2) Illustration in the bar chart in Fig. 25, it can be seen that: compared with the floor without laying system, when the upper surface of the floor laying system has a floor airborne sound insulation performance is from 45 dB (level 7) to 47 dB (level 7), as well as when the floor on the upper and lower surfaces of the laying of the system is 20 mm, the floor airborne sound insulation performance of 50 dB (level 8), to meet the current standards of the "Code for the Design of Sound Insulation of Civil Buildings" (GB50118-2010)^22^ high limit (floor airborne sound insulation ≥ 50 dB) requirements. It can be seen that the sound insulation, thermal insulation carbon porous ball foam system in the upper floor when the floor airborne sound insulation is improved but not obvious, the system under the combination of the lower and upper, the floor airborne sound insulation performance has been improved to a certain extent, to improve the airborne sound insulation amount of 5 dB. This is due to the laying of the system in the floor under the surface of the floor. This is due to the activated carbon fibre, wood chip powder, high temperature glass beads, ultra-fine inorganic rock wool fibre and other materials foamed to form particles of acoustic ball after the surface of the fine holes, these holes have a very good sound-absorbing properties. So that the noise in the countless times of the process of impact with the holes gradually weakened to enhance the airborne acoustic insulation performance of the floor.
(3) It can be seen in the bar chart in Fig. 25 that, compared with the external wall without laying system, the performance of airborne sound insulation of the external wall is improved from 44 dB (level 6) to 47 dB (level 7) when the system is laid on the external surface of the external wall, which is according to the "Acoustic Building and Building Component Sound Insulation Measurement Part 5 On-site Measurement of Airborne Sound Insulation of Exterior Wall Components and Exterior Walls" (GB-T 19889.5-2006)^25^. The loudspeaker simulates the noise source, and when the noise spreads to the external surface of the external wall, the carbon fibre porous ball, wood chip powder, high temperature resistant glass beads, ultrafine inorganic rock wool fibre, and other materials of the sound insulation and thermal insulation carbon porous ball foaming system attenuate the noise, which makes the noise transmitted into the room smaller. And plays a certain sound insulation effect, but the experiment is also subjected to the interference of the uncontrollable factors of the external wall windows, doors, and other uncontrollable factors.

### Analysis of the results of the thermal performance of floor slabs and external walls

**Figure 28:**
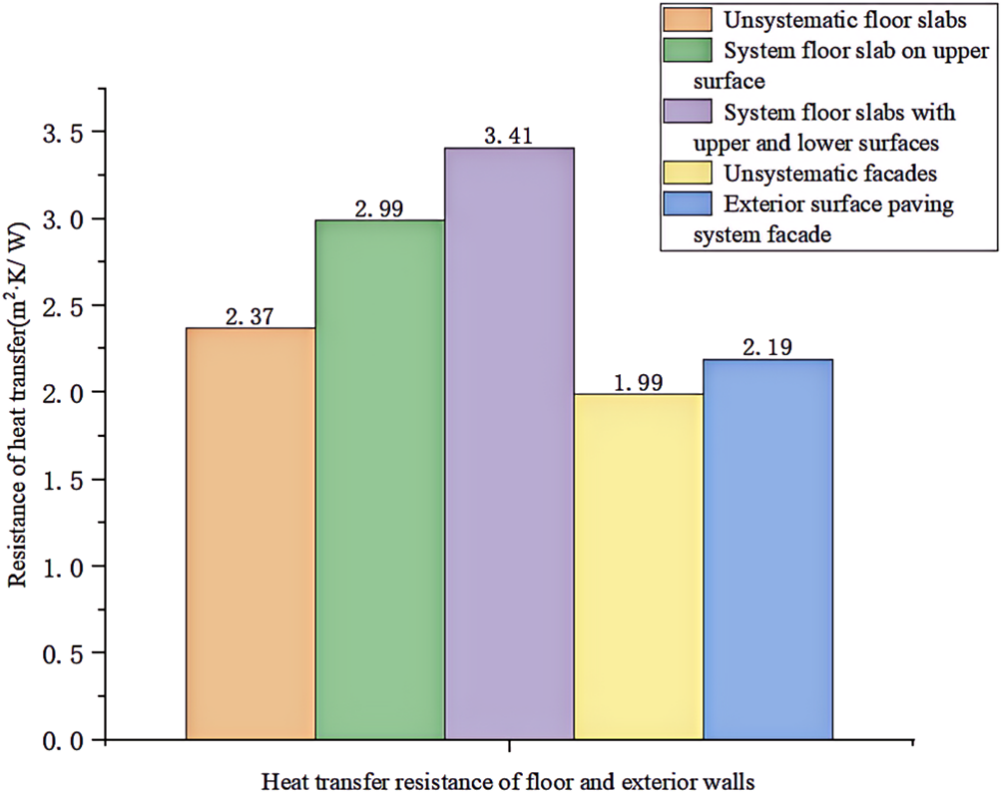
Comparison of thermal performance results for floor and exterior walls.

1. As can be seen from the bar chart in Fig. 28, comparing the results of the thermal performance of the floor slab and external wall compared with the floor slab without a laying system: when a 20 mm sound insulation and heat preservation carbon porous sphere foam system is laid on the upper surface of the floor slab, the thermal resistance of the floor slab, *R*_*L*_, is increased from 2.37 m^2^ K/W to 2.99 m^2^ K/W, and *R*_*L*_ will be increased to 3.41 m^2^ K/W. If it is laid on both the upper and lower surfaces of the floor slab at the same time, the system has better sound insulation performance than the floor slab with the same thermal resistance. This test result proves that the system has better sound insulation performance, thanks to the fact that the system has HB polymer cementitious JS-II type, activated charcoal fibre, wood chip powder, 9003-35-4 high-temperature-resistant phenol-formaldehyde resin, high-temperature-resistant glass beads, ultrafine inorganic rock wool fibre, high-foaming elastic material, etc. All of which are high-quality building thermal insulation materials with low thermal conductivity, which proves their high-quality thermal insulation performance.
2. As can be seen from the comparative histogram of the thermal performance results in Fig. 26, the thermal resistance *R*_*Q*_ of the external wall increases by 0.2 m^2^ K/W. When the system is laid on the external surface of the external wall compared with the external wall without the laying system, as analysed in principle in 2.2(1) above.

## Conclusions

In this paper, the design of an acoustic insulation, thermal insulation carbon porous ball foam system design, and the floor plus the outer wall made of variety of different structural combination. Based on different layering methods, the following main conclusive remarks are as follows: The comparative analysis of the test results of the different laying methods of the floor acoustic sound insulation performance, the floor thermal performance, the airborne acoustic sound insulation performance of the wall, and the thermal performance of the external wall:

1. The system, due to the modulus of elasticity (Pa) of the better material for the foam after the intervention, The system as a floating floor sound insulation material, can effectively reduce the upper surface of the floor impact to the vibration of the floor, as well as a vertical sound insulation piece that can effectively block the floor and the wall columns and columns between the occurrence of the "resonance" situation. However, there is no obvious improvement of the sound insulation when the system is laid on the lower surface of the floor. The reason is that the system has no contact with the impact object and cannot directly block the impact, so to improve the sound insulation performance of the floor, the system needs to be laid on the surface of the floor (in contact with the impact object). At the same time, needs to be inlaid in the vertical "gap" in the contact between the floor and the wall columns. "In the vertical contact between the floor slab and the wall column, as a vertical sound insulation piece.
2. The surface of the foam particle acoustic ball in the system has micropores, which, after theoretical and experimental tests, showed that it has good sound absorption performance. So, the system can be used as a floor pavement system, and can be a good way to increase the airborne sound insulation performance of the floor. At the same time, as the external wall of the external pavement system, it can also play a role in the absorption of outside noise to enhance the airborne sound insulation performance of the external wall.
3. The high-quality building thermal insulation material with low thermal conductivity added to the system effectively enhances the thermal insulation performance of the system. So, any combination of the system with the floor slabs and the external wall structure can effectively increase the thermal resistance value of the floor slabs and the external wall system and enhance their thermal insulation performance.
4. Vibration damping, sound insulation, thermal insulation porous ball foaming system in the existing research basis to promote the development of the field of green building is mainly embodied in the following aspects: ① to enhance the energy-saving performance of the building: through the use of lightweight, highly efficient thermal insulation of porous materials, you can improve the thermal insulation properties of the building to reduce energy consumption and reduce carbon emissions. Porous spheres have lightweight, acoustic, thermal insulation and other characteristics, which help to improve the building's energy-saving standards; ② Enhancement of the building's acoustic performance: the mosaic of porous spheres, the unique structure of the material can effectively absorb and reduce the spread of noise, reduce vibration, and improve the comfort of the living and working space. Relevant engineering experiments have been fully confirmed; ③ Promote the industrialisation and standardisation of green buildings: the scientific laying method and standardised construction of the porous foam system can improve the convenience and efficiency of construction. Meanwhile, the future standardised production and application of the system in building exterior walls and floor slabs will help improve the efficiency and quality of building construction and reduce the generation of construction waste.

## Data Availability

The datasets generated and/or analysed during the current study are not publicly available but are available from the first author Dr. Hua Shi on reasonable request.
